# A Review on Environmental Contaminants-Related Fertility Threat in Male Fishes: Effects and Possible Mechanisms of Action Learned from Wildlife and Laboratory Studies

**DOI:** 10.3390/ani11102817

**Published:** 2021-09-27

**Authors:** Sayyed Mohammad Hadi Alavi, Sepideh Barzegar-Fallah, Parastoo Rahdar, Mohammad Mahdi Ahmadi, Mina Yavari, Azadeh Hatef, Mahdi Golshan, Otomar Linhart

**Affiliations:** 1 School of Biology, College of Science, University of Tehran, Tehran P.O. Box 14155-6655, Iran; sepidehbarzegar95@gmail.com (S.B.-F.); p.rahdar@ut.ac.ir (P.R.); mahditerm@gmail.com (M.M.A.); mina.yavari4927@ut.ac.ir (M.Y.); 2 Toxicology Centre, University of Saskatchewan, Saskatoon, SK S7N 5B3, Canada; azadeh.hatef@usask.ca; 3 Iranian Fisheries Science Research Institute, Agricultural Research, Education and Extension Organization, Tehran P.O. Box 15745-133, Iran; mahdigolshan@yahoo.com; 4 South Bohemian Research Center of Aquaculture and Biodiversity of Hydrocenoses, Faculty of Fisheries and Protection of Waters, University of South Bohemia in České Budějovice, 389 25 Vodňany, Czech Republic; Linhart@frov.jcu.cz

**Keywords:** fertility endpoints, industrial pollutants, pesticides, pharmaceuticals, sperm quality

## Abstract

**Simple Summary:**

Public concern regarding environmental contaminants (ECs)-related reproductive disorders has increased due to increasing global rates of infertility. All kinds of ECs are on rise rapidly in developing and industrializing low- and middle-income countries. The aquatic environments throughout the world are repositories for enormous amounts of ECs. As the biology of the reproductive system is highly conserved in vertebrates, wildlife or laboratory studies on fish provide significant information to establish a detailed risk assessment, and to identify novel or more sensitive endpoints for ECs-related reproductive disorders. The adverse effects of ECs on endocrine regulation of reproduction in male fishes have been extensively studied and reviewed; however, our knowledge on the effects and mechanisms of action of ECs on determinants of male fertility is limited. The present study is a state-of-the-art comprehensive review on the ECs-related fertility threat in male fishes with emphasis on the ECs effects on sperm production, morphology, genome, and motility kinetics. After a brief introduction to reproductive biology, fertility indicators, and determinants of fertility in male fishes, wildlife evidences for reproductive disorders were reviewed in fishes from the polluted aquatic environment. The laboratory studies show that ECs detected in aquatic environment are capable of causing fertility threat at environmentally relevant concentrations associated with a decrease in fertility determinant(s). This study suggests an urgent need to better elucidate mechanisms through which ECs affect sperm functions to cause fertility threat.

**Abstract:**

Increasing global rates of diminished fertility in males has been suggested to be associated with exposure to environmental contaminants (ECs). The aquatic environments are the final repository of ECs. As the reproductive system is conserved in vertebrates, studies on the effects of ECs on fertility endpoints in fishes provide us with valuable information to establish biomarkers in risk assessment of ECs, and to understand the ECs-related fertility threat. The aim of the present review was to evaluate associations between ECs and fertility determinants to better understand ECs-related male fertility threat in male fishes. Wildlife studies show that the reproductive system has been affected in fishes sampled from the polluted aquatic environment. The laboratory studies show the potency of ECs including natural and synthetic hormones, alkylphenols, bisphenols, plasticizers, pesticides, pharmaceutical, alkylating, and organotin agents to affect fertility determinants, resulting in diminished fertility at environmentally relevant concentrations. Both wildlife and laboratory studies reveal that ECs adverse effects on male fertility are associated with a decrease in sperm production, damage to sperm morphology, alternations in sperm genome, and decrease in sperm motility kinetics. The efficiency of ECs to affect sperm quality and male fertility highly depends on the concentration of the contaminants and the duration of exposure. Our review highlights that the number of contaminants examined over fertility tests are much lower than the number of contaminants detected in our environment. The ECs effects on fertility are largely unknown when fishes are exposed to the contaminants at early developmental stages. The review suggests the urgent need to examine ECs effects on male fertility when a fish is exposed at different developmental stages in a single or combination protocol. The ECs effects on the sperm genome are largely unknown to understand ECs-related inheritance of reproductive disorders transmitted to the progeny. To elucidate modes of action of ECs on sperm motility, it is needed to study functional morphology of the motility apparatus and to investigate ECs-disrupted motility signaling.

## 1. Introduction

Global rates of environmental contaminants (ECs)-related reproductive disorders have been increasing over the past 50 years. In human beings, the incidences of testicular dysgenesis syndrome, including hypospadias (urethra opens on the underside of the penis instead of the tip), cryptorchidism (one or both testes not descended into the scrotum), testicular cancer, low semen quality, and infertile men, show global increases associated with ECs [1,2,3,4,5,6,7,8,9]. Landrigan et al. [10] reported that all kinds of ECs are all on the rise rapidly in developing and industrializing low-income and middle-income countries. The public concern regarding ECs-related reproductive disorders was originally linked to observations of reduced fertility, birth defects, and sexual developmental disorders in wildlife [11]. For over 30 years, the World Health Organization (WHO), National Institute of Health (NIH, USA), European Food Safety Authority, and the other organizations composed of working groups of experts in endocrinology, risk assessment, and toxicology, have conducted studies to examine the adverse effects of ECs on reproduction in humans and wildlife. These studies have shown that there are about 800 natural and man-made chemicals known or suspected to interfere with physiological and endocrinological regulation of reproduction [12]. However, our knowledge on the ECs-related hormonal dysfunctions that cause diminished fertility is limited to a small fraction of these chemicals. To reduce ECs-related fertility threat in males, it is critical to identify the contaminants that interfere with determinants of fertility, including sperm production, morphology, genome, and motility, and to characterize their modes of action on reproductive endocrine system. In this regard, interdisciplinary efforts that combine knowledge from wildlife, experimental animals, and human infertility clinics are needed to provide a more holistic approach for ECs-related reproductive disorders and fertility threat.

The aquatic environment is at greatest risk from pollutants since all chemicals will eventually find themselves in the rivers, lakes, and oceans as the final repository [13]. As biology of reproduction is highly conserved in vertebrates [14,15,16], studies on fishes as model organisms provide significant information to establish a detailed risk assessment and to establish novel or more sensitive endpoints for ECs-related fertility threat. Frequent clear evidences show reproductive disorders in fishes from polluted aquatic environments (see Section 4). The adverse effects of ECs on endocrine regulation of reproduction in male fishes have been extensively studied and reviewed in laboratory studies [13,17,18,19,20,21,22,23,24,25]. In contrast, our knowledge to understand whether ECs-disrupted hormonal functions result in diminished fertility is poor. To answer, it is critical to uncover the adverse effects of ECs on sperm production, morphology, genome, and motility kinetics as key determinants of fertility.

We have recently reviewed the toxicity of ECs on sperm morphology and motility in fishes, in vitro [26]. The review showed that ECs, in a dose-dependent manner, cause damage to sperm morphology and interfere with sperm energetics and motility kinetics, and thus affect male fertility. However, significant decreases or complete suppression of sperm motility and fertilizing ability occurred mostly at concentrations considerably higher than those reported in the aquatic environment or exceeding the WHO recommended limits for surface waters. Recently, Carnevali et al. [27] and Golshan and Alavi [25] suggested that ECs are capable of affecting sperm quality in fishes associated with alternations in hormonal functions of hypothalamus–pituitary–testis (HPT). These reviews have mostly focused on studies that show the adverse effects of ECs on sperm functions using in vitro approaches, and, moreover, the effects of a few ECs have been reviewed.

The present study is a state-of-the-art comprehensive review on the ECs-related fertility threat in male fishes with emphasis on the adverse effects of ECs on determinants of fertility, including sperm production, morphology, genome, and motility kinetics. After a brief introduction to reproductive biology, fertility indicators, and determinants of fertility in male fishes, wildlife evidences for reproductive disorders are reviewed in fishes from the polluted aquatic environment. To understand whether reproductive disorders in wildlife is associated with ECs, we review laboratory studies in which the adverse effects of particular ECs are studied on determinants of fertility, in vivo. Finally, we discuss urgent needs to better elucidate mechanisms through which ECs affect sperm functions to cause fertility threat. The present review provides us with valuable information to understand ecotoxicological impacts of ECs on fish fertility, which can be useful to establish biomarkers in ECs risk assessment.

## 2. An Introduction to Reproductive Biology in Male Fishes

It is essential to review reproductive biology, fertility indicators, and determinants of fertility in male fishes before delving into the ECs-related fertility threat. These provide the basic information to better understand multiplicity of sites through which ECs interfere with fertility. To clarify the terminology, “semen” refers to seminal plasma and sperm and “sperm” refers to sperm cells in the present review.

### 2.1. Anatomy of Reproductive Organ

**Figure 1:**
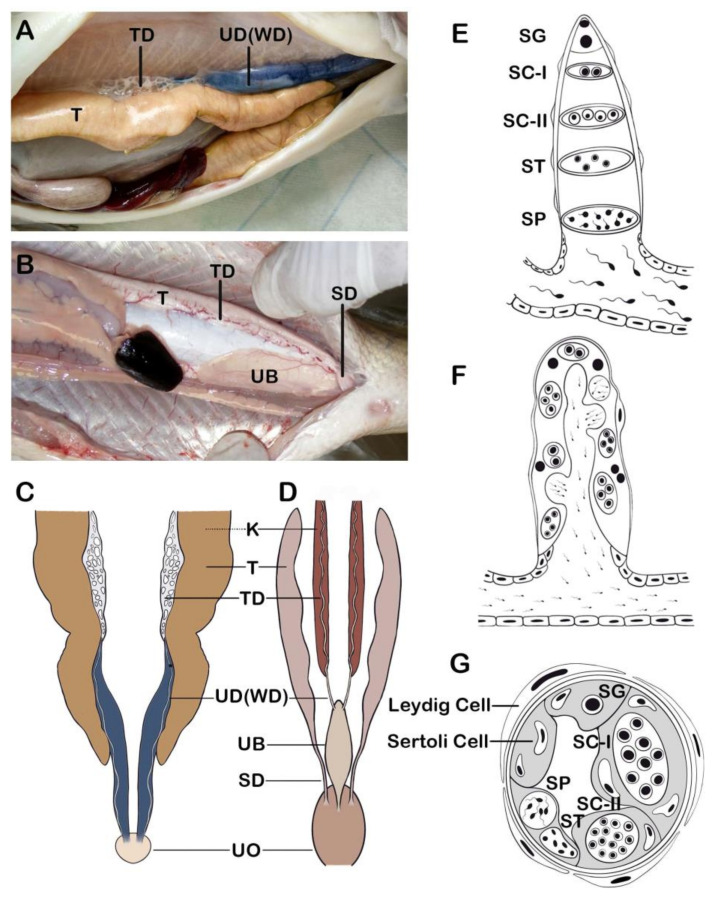
Reproductive system in male fishes. Panels (**A**,**C**) show the anatomy of the reproductive system in primitive fishes (sturgeons). Sperm is released from the testes into the testicular ducts, which pass the kidney. At spawning, semen is released into the aquatic environment through the urinary ducts opened into the urogenital opening (UO). Panels (**B**,**D**) show the anatomy of the reproductive system in bony fishes (teleosts). Sperm is released from the testes into the testicular ducts. At spawning, semen is released into the aquatic environment through the sperm ducts opened into the UO. Panels (**E**,**F**) are schematic of the tubular testis and lobular testis, respectively. Panel G shows testicular compartments in fishes. K, kidneys; SD, sperm duct; SC-I, primary spermatocyte, SC-II, secondary spermatocyte; SG, spermatogonia; SP, spermatid; SZ, sperm; T, testis; TD, testicular duct; UB, Urinary bladder; UD (WD), urinary duct (Wolffian duct). The panels are modified from Grier [30], Nagahama [32]; Alavi et al. [28] and Dzyuba et al. [29]. The photo of panel A is courtesy of Associate Professor Borys Dzyuba from the sterlet (*Acipenser ruthenus*). The photo of panel B is from S. M. H. Alavi from the Northern pike (*Esox Lucius*). Panels C-G credits: © S. Barzegar-Fallah.

In general, the male reproductive organ consists of a paired testes, the testicular duct, and the sperm duct in fishes [28,29] (Figure 1). In some primitive fishes (such as sturgeons), the testes release sperm into the testicular ducts, which pass the kidneys. At spawning, semen is released into the aquatic environment through the urinary ducts opened into the urogenital opening (Figure 1A,C). In most bony fishes (teleosts), neither testicular ducts nor sperm ducts attach to the kidneys. The sperm is released from the testes into sperm ducts where seminal plasma is secreted. At spawning, semen is released into the aquatic environment through the sperm ducts opened in the urogenital opening (Figure 1B, bookmark0D).

The testes are divided into the “tubular type” and the “lobular type” according to the distributions of spermatogonia in the seminiferous region [30,31,32,33] (Figure 1E, bookmark0F).

In the tubular type, as spermatogonia divide and enter in meiosis, the cysts migrate towards the region of the spermatic ducts located in the central region of the testis, where the cysts open to release sperm (Figure 1E). This type of testicular arrangement is found in zebrafish (*Danio rerio*) and guppy (*Poecilia reticulate*).

In the lobular type, the testis is composed of numerous lobules that are separated from each other by a thin layer of fibrous connective tissue, and spermatogonia are spread along the germinal compartment throughout the testis. The cysts do not migrate or become displaced during their development, and sperm is released into the lobular lumen (Figure 1F). This type of testicular arrangement is found in Japanese medaka (*Oryzias latipes*), common carp (*Cyprinus carpio*), goldfish (*Carassius auratus*), and rainbow trout (*Oncorhynchus mykiss*).

The testicular compartment contains Sertoli cells, Leydig cells, blood/lymphatic vessels, macrophages and mast cells, and neural and connective tissue cells (Figure 1G). The Leydig and Sertoli cells are involved in biosynthesis of steroid hormones to regulate sperm production and maturation.

The testicular ducts are located adjacent to the testes, which continue into the sperm ducts on the ventral sides. Testicular and sperm ducts possess very similar structural and enzyme-histochemical characteristics, and play key roles in nutrition of sperm, storage of sperm, synthesis of steroids, secretion of proteins and enzymes, and formation of the seminal plasma [34,35]. Maturation of sperm to acquire potential for motility and fertilizing ability occurs in the sperm ducts [36,37].

### 2.2. Spermatogenesis

Sperm is produced from spermatogonia following divisions [15,38,39]. During the process of spermatogenesis, diploid spermatogonia type A divides mitotically to produce diploid spermatogonia type B. The final mitotic division of spermatogonia type B produces diploid primary spermatocytes that undergo the first meiotic division to form haploid secondary spermatocytes. The second meiotic division produces haploid spermatids that transform into the flagellated sperm.

### 2.3. Sperm Morphology

**Figure 2:**
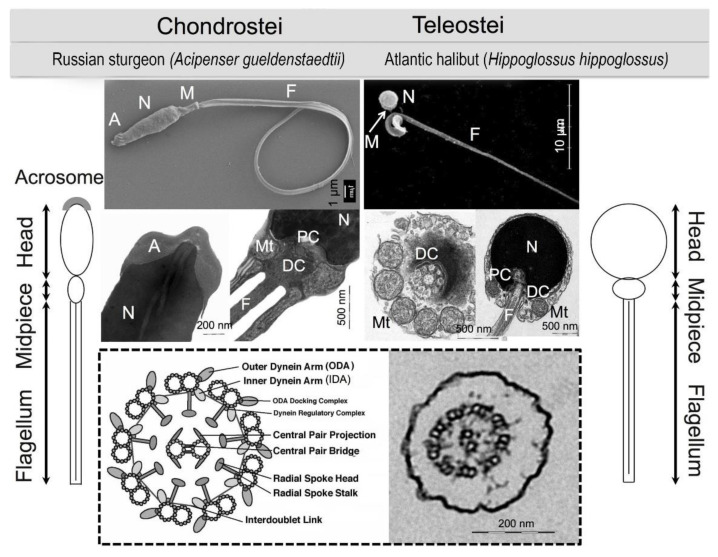
Sperm morphology in primitive (chondrostei) and bony (teleostei) fishes. Sperm is composed of a head (nucleus, N), midpiece (M) and flagellum (F). In chondrostei fishes (such as sturgeons), there is an acrosome (A) at the top of the head of sperm. The ultrastructure compartments of sperm are similar between chondrostei and teleostei fishes: DC, distal centriole; PC, proximal centriole; Mt, mitochondria. The structure of the motility apparatus called “axoneme” is highly conserved, and possesses the typical 9 + 2 microtubule structure of cilia surrounded by plasma membrane. The electron micrographs are selected from the Russian sturgeon (*Acipenser gueldenstaedtii*) [47], and Atlantic halibut (*Hippoglossus hippoglossus*) sperm [48]. The schematic of the axoneme is from Inaba [49].

Sperm is differentiated into a head, midpiece, and flagellum in fishes [40,41,42] (Figure 2). The head of sperm contains DNA for transferring a haploid set of the chromosomes into the oocyte upon fertilization. Mitochondria and proximal and distal centrioles are located in the midpiece. Mitochondria deliver energy that is required for the beating of the sperm motility apparatus with a “9 + 2” structure called the “axoneme” [43,44]. Both proximal and distal centrioles consist of nine peripheral triplets of microtubules. The distal centriole forms the basal body of the axoneme. Sperm is acrosomeless in teleostean fishes, while it possesses acrosome in primitive fishes, including hagfish and sturgeons [45,46].

### 2.4. Sperm Physiology

The seminal plasma is a product of Sertoli cells, testicular ducts, and sperm ducts, and its composition is different among fishes that may reflect species variations. The main role of seminal plasma is to create an optimal environment for the storage of sperm during maturation in the sperm ducts. Seminal plasma maintains sperm viability, motility, and fertilizing ability, and protects sperm against damage caused by proteolytic or oxidative attacks [50,51].

### 2.5. Sperm Motility

**Figure 3:**
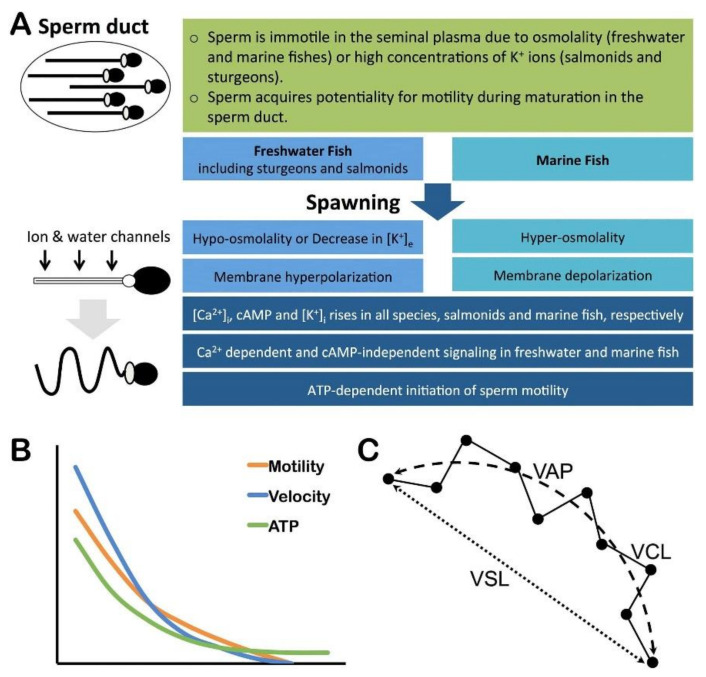
Sperm motility signaling and kinetics in fishes. Panel (**A**) summarizes sperm motility signaling in fishes. Sperm is immotile in the sperm ducts and seminal plasma. At spawning, a hypo- osmolality accompanied by K+ efflux or hyper-osmolality accompanied by water efflux trigger sperm motility activation in freshwater and marine fish species, respectively. Activation of ATP- dependent sperm motility initiation is associated with an increase in intracellular calcium ([Ca2+]*i*) ions in all fish species and an increase in intracellular potassium ([K+]*i*) ions in marine species, while cyclic adenosine monophosphate (cAMP) remains unchanged. However, demembranated sperm of salmonids requires cAMP for axonemal beating. Panel (**B**) shows sperm motility kinetics in fishes. After initiation of sperm motility, percentage of motile sperm and sperm velocity decrease rapidly in both freshwater and marine fishes due to depletion of adenosine triphosphate (ATP) content. Panel (**C**) is a schematic representing various sperm velocity parameters analyzed by a computer-assisted sperm analysis. The curvilinear velocity (VCL) is the velocity along the trajectory of sperm head. The straight line velocity (VSL) is the straight line distance between the start and end points of the track divided by the duration of the movement. The angular path velocity (VAP) is the velocity along a derived smoothed path.

Sperm is generally immotile in the seminal plasma and the sperm ducts of fishes, and motility is triggered upon discharge into the aquatic environment (Figure 3A). In most freshwater and marine fishes, osmolality of the seminal plasma is the key factor to maintain sperm in the quiescent state in the sperm ducts [52]. In some freshwater fishes, including Salmonidae and Acipenseridae, high concentrations of potassium (K+) ions inhibits sperm motility in the seminal plasma [53,54,55]. At spawning, a hypo-osmotic and a hyper-osmotic signal is necessary for initiation of sperm motility in freshwater and marine fishes, respectively [42,56,57,58,59]. Changes of osmolality around sperm accompanied by K+ efflux in freshwater fishes and water efflux in marine fishes trigger sperm motility signaling. Activation of sperm motility is associated with an increase in intracellular pH and calcium (Ca^2+^) ions in both freshwater and marine fishes, while cyclic adenosine monophosphate (cAMP) remains unchanged. However, studies show that demembranated sperm of salmonid and sturgeon fishes require cAMP for the axonemal beating [60,61,62]. In some marine fishes, it has been shown that 17,20*β*,21-trihydroxy-4-pregnen-3-one (17,20*β*-P) is capable to induce sperm hypermotility by increasing cAMP and intracellular Ca^2+^ through a membrane progesterone receptor [63,64].

After initiation of sperm motility in the aquatic environment, duration of motility is very short in fishes from a few seconds to several minutes or hours depending on the species [28,29,51,59,65,66]. The inter-species differences probably depend on the capacity of the sperm to restore intracellular ATP and creatine phosphate concentrations [67,68]. Once sperm motility is initiated, the percentage of motile sperm and sperm velocity rapidly decrease, which are associated with a large, but not complete depletion of ATP [46,59,66] (Figure 3B). Fish sperm can regenerate ATP from phosphocreatine and ADP; however, this ATP regeneration system does not prevent the precipitous decline in ATP levels during motility [69,70,71].

## 3. Fertility Indicators and Assessments in Fishes

**Figure 4:**
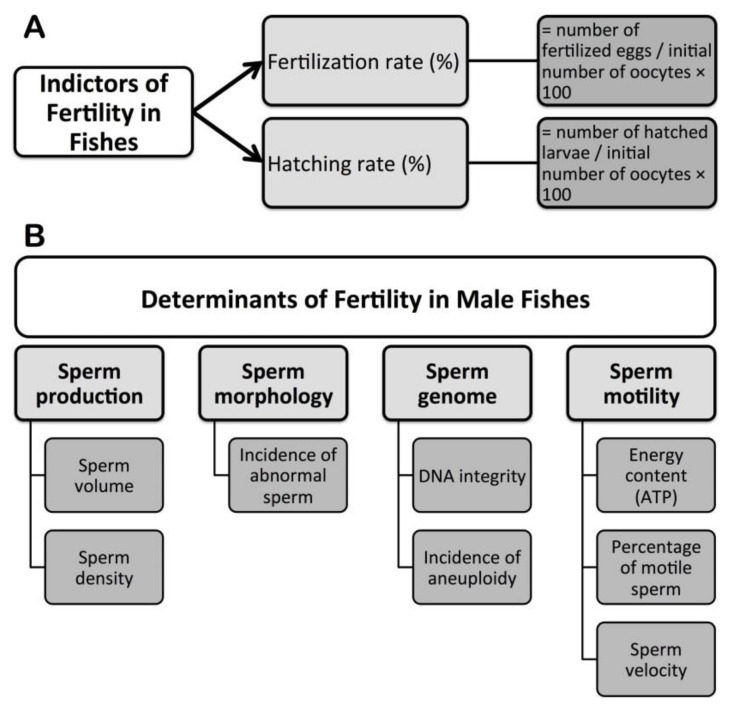
Indicators and determinants of fertility in male fishes. Fertilization success is assessed by fertilization rate or hatching rate (**A**). Sperm production, morphology, genome and motility are key determinants of fertility in male fishes (**B**).

Fertilization and hatching rates are calculated to assess fertility in fishes (Figure 4A). The fertilization rate is the percentage of oocytes that become fertilized upon spawning or artificial insemination, and is calculated as number of fertilized eggs/initial number of oocytes × 100. Successful fertilization depends on onset of release of sperm from males and ova from females [28,72]. During the short period of motility, sperm must penetrate the oocyte through a funnel called the “micropyle” to fertilize it [32,73]. A fertilized egg can be easily identified by the presence of a multi-cellular blastodisc (cleavage), which occurs from several hours to a few days post fertilization and depends on fish species and environmental factors including temperature. The hatching rate is the percentage of hatched larvae, and calculated as number of hatched larvae/initial number of oocytes × 100. Once embryonic development is completed, larvae hatch [74,75].

## 4. Determinants of Fertility in Male Fishes

Analyses of sperm production, morphology, genome, and motility kinetics are basically important to assess fertility in male fishes (Figure 4B).

### 4.1. Sperm Production

Frequent studies have shown that fertilization rate positively correlates with sperm volume, sperm density, number of sperm per oocyte, and density of sperm in the water during fertilization [76,77,78,79,80,81,82,83]. One can weigh semen mass, measure semen volume, or count sperm density to evaluate sperm production.

### 4.2. Sperm Morphology

There is a species-specific relationship between the head size of sperm and diameter of micropyle in fishes [49,73,74]. This indicates that sperm of one species can penetrate only into the oocyte of similar species. A change in the size of sperm head is a mirror of the size of nucleus [84,85,86]. It has also reported that sperm with a smaller head can move faster than those with a larger head [86,87,88]. Additionally, both positive and negative correlations have been reported between the length of flagellum and the sperm velocity [86,87,88,89,90,91]. These suggest that alternations in the size of sperm head and length of flagellum can result in diminished fertility by affecting sperm penetration into the oocytes or sperm motility performance. Various microscopic techniques including scanning and electron microscopy are valuable methods to assess sperm morphology [43,45,92].

### 4.3. Sperm Genome

Upon fertilization, sperm with a haploid number of chromosomes transmit a parental genome to the next generation. Alternation of chromosome material, Y chromosome deletion, and ploidy level are among factors that affect fertility. The integrity of sperm DNA correlates with fertilization and embryonic development in fishes [93,94]. Fertility threat has been frequently reported in polyploid fish, which were associated with failure of testicular development [95], enlarged head size making penetration of sperm through a normal-sized micropyle difficult [96,97], reduced sperm production [84,98], and increased abnormal sperm with malformation of the head, mitochondria, and flagellum resulting in decreasing motility and velocity [84,99]. One can assess the integrity of DNA using a comet assay, sperm chromatin structure assay, or terminal deoxynucleotidyl transferase dUTP nick end labelling assay (TUNEL) [100,101]. Chromosome number and DNA content can be counted or assessed using a flow cytometry, respectively [84,86,95].

### 4.4. Sperm Motility Kinetics

Duration of sperm motility, percentage of motile sperm, and sperm velocity are key determinants for fertility in male fishes [28,79,102]. It has been shown that sperm with faster movement and a longer period of motility have more chance to approach an oocyte to fertilize it [78,80,103,104]. In addition, it has been suggested that sperm velocity and the duration of motility are positively correlated with ATP content of sperm [59,66]. A computer-assisted sperm analysis (CASA) provides a valuable tool to assess sperm motility kinetics [105,106,107]. Percentage of sperm motility is evaluated by counting the number of motile sperm and total number of sperm. The sperm velocity is the distance between the starting and ending points of the motility track divided by the time spent for this movement. Based on sperm head positions during the period of motility, various sperm velocity parameters are identified, including curvilinear velocity (VCL, the velocity along the sperm head trajectory), straight line velocity (VSL, the straight line distance between the start and end points of the sperm head trajectory), and the angular path velocity (VAP, the velocity along a derived smoothed path) (Figure 3C).

## 5. Wildlife Evidences for Environmental Contaminants (ECs)-Related Fertility Threat in Male Fishes

Wildlife studies have been frequently conducted to investigate reproductive disorders in male fishes inhabiting the aquatic environment that receive bleached kraft pulp mill effluent, municipal wastewater effluent, sewage treatment plant effluent, wastewater treatment plant effluent, and agricultural and industrial runoffs. Various types of hormonal mimic or genotoxic ECs have been detected including natural and synthetic hormones, pesticides, metals, industrial chemicals (nonylphenol, alkylphenol, bisphenols, phthalates, polychlorinated biphenyls, hexachlorocyclohexane and hexachlorobenzene), and phytosterols. These studies have shown various reproductive disorders in males from the polluted aquatic environments (Table 1).

Delay in sexual maturity, decrease in gonadosomatic index (gonad mass/body mass × 100) and secondary sexual characteristics, and increases in female-biased sex ratio and intersex (occurrence of oocytes in the testes) are predominant reproductive disorders reported in fishes from polluted aquatic environments [108,109,110,111,112,113,114,115,116,117,118,119,120,121,122,123,124,125]. The incidence of intersex has been shown to be mostly associated with increased circulating levels of vitellogenin (Vtg) or 17*β*-estradiol (E_2_) [111,113,115,117,118,123,124,126,127,128,129,130].

Compared to a number of studies that reported incidence of intersex and the size of testis, there are a few studies that investigated sperm quality. These studies demonstrate the adverse effects of ECs to decrease number of spermiating males, sperm production (volume and density), viability, motility, velocity, and fertilizing ability, and to increase number of abnormal sperm and sperm chromosome breakage [115,117,121,131,132,133,134,135]. Decreases in sperm production have been shown to be mostly associated with decreases in circulating androgen levels (testosterone (T) or 11-ketotestosterone (11-KT)), which lead to disorders in testicular development [115,121,122,129,130,134,135,136,137]. It has been also shown that decreases in fertilizing ability of fish from polluted aquatic environments were associated with decreases in sperm production, motility, or velocity [115,132,134].

Taken together, wildlife studies reveal that ECs are capable of impairing spermatogenesis and sperm development in fishes. Subsequently, morphological damage to sperm, abnormality of the sperm genome, decrease in sperm production, and reduction of sperm motility kinetics result in diminished fertility in male fishes. It is worth to note that wildlife studies also showed ECs-related fertility threat in male fishes which were associated with disruption of hormonal functions of the hypothalamus–pituitary–testis axis that regulate spermatogenesis and sperm development (Table 1).

**Table 1:** Wildlife evidences for environmental contaminants-related reproductive disorders in male fishes.

Region	Fish Species	Contaminants	Reproductive Endpoints	Description	Authors
Jackfish Bay, North Shore of Lake Superior, Canada	White sucker	BKME that includes resin acid	Delayed sexual maturation Decreased testicular size Reduced secondary sexual characters Decreased LH, T, 11-KT, and 17,20β-P levels Failed to increase 17,20β-P levels in response to stimulation of spermiation by sGnRH-A	Polluted site: Jackfish bay Reference site: Mountain bay Sampling time: May–Aug 1988 (Munkittrick et al. 1991); Aug 1988, Aug 1989 and Sep 1990 (Munkittrick et al. 1992); May 1990 and 1991 (Van Der Kraak et al. 1992)	[109,110,138]
Estuarine, UK	Flounder	Sewage effluents	No difference in GSI, increased Vtg levels at all polluted sites Intersex at the Mersey estuary (17%)	Polluted sites: Tyne, Crouch, Thames and Mersey estuaries Reference site: Alde River Sampling time: Sep–Dec 1996	[111]
The Moselle and the Rhone Watershed, France	Chub	Metals and organic chemicals	No difference in GSI Increased Vtg levels at Igney Necrotic sperm cells observed at Igney	Polluted site: Igney (the Moselle river) Reference site: Saillans (the Drome river) Sampling time: June	[139]
Saint Louis, Missouri, USA	Shovelnose sturgeon	Chlordane, *p,p’.* DDE, PCBs	Intersex (29%)	Polluted site: Downstream of St. Louis, Prairie de Rocher, IL Reference site: Upstream of St. Louis, Davenport, IA Sampling time: Autumn	[112]
Tokyo Bay, Japan	Flounder	Sewage effluents that include Alkylphenol polyethoxylates and NP	Decreased GSI in males with ovo-testis Increased Vtg levels Intersex (15% of individuals)	Polluted site: Tokyo Bay, Japan Reference site: Shiriuchi, Hokkaido, Japan Concentrations (μg/L): Alkylphenol polyethoxylates (37.5), Nonylphenol: (0.2–1.1) Sampling time: Jan 1997–May 1998	[113]
The Po River, Italy	Barbel	Not determined	Intersex (50%)	Polluted site: Downstream of the Po River Reference site: Upstream of the Po River Sampling time: April 1999	[114]
Nene and Aire Rivers, UK	Roach	STWs	Decreased GSI Intersex at Nene and Aire (100%) (1–4% intersex at reference site) Delayed spermatogenesis Increased Vtg, T, and E_2_ levels in intersex males compared to normal and to intersex males of reference sitesNo difference in 11-KT levels between intersex males of polluted sites and reference sites Decreased number of spermiating males Decreased sperm volume and density Decreased sperm motility and velocity Decreased fertilizing ability of sperm	Polluted sites: Nene (Northamptonshire) and Aire (Yorkshire) Reference cites: Royal Canal (Ireland), Grantham Canal (Leicestershire) and a spring-fed lake (Lake Wartnaby, Leicestershire) Sampling time: October 1995 and 1996; May 1998, 1999, and 2000	[115,132]
The St. Johns River	Largemouth bass	DDT and its metabolites, PCBs, high and low molecular weight PAHs, cyclodiene and chlorinated pesticides	At pre-spawning season (Sep 1996): Increased E_2_ levels at polluted sites Decreased 11-KT levels at Palatka and Julington Creek At Spawning season (February 1997): Decreased GSI at Palatka, increased E_2_ levels at Palatka and Green Cove, decreased E_2_ levels at Julington CreekDecreased 11-KT levels at all polluted sites	Polluted sites: Downstream of the St. Johns River: Palatka (North of Welaka), Green Cove (North of Palatka) and Julington Creek (North of Green Cove). The Palatka and Green Cove sites are representative of urban, industrial, and agricultural development. The Palatka site is near to a paper mill plant. The Julington Creek site receives discharges of wastewater and runoff from recreational boating marinas Reference site: Upstream of the St. Johns River Sampling time: September 1996 and February 1997	[116]
The Anoia River, Spain	Carp	STW that includes estrogenic compounds	Decreased testicular size Occurrence of testicular atrophy Intersex (19%) Decreased T levels, Increased Vtg levels	Polluted sites: Downstream of the Anoia River Reference site: None Sampling time: January and March 2000	[126]
The St. Lawrence River, Montreal, Canada	Spottail Shiner	STW that includes xenoestrogens	Intersexes at Îles de la Paix, Île Dorval, Îlet Vert and Île Beauregard (2.6, 15, 31, and 27, respectively) Increased Vtg mRNA levels at Îlet Beauregard, Îlet Vert, and Île Saint-Ours compared to the Îles de Boucherville, Île Dorval, Îles de la Paix, and Ottawa river Delayed spermatogenesis at Îlet Vert and Île Beauregard Decreased sperm density, motility, and velocity at Îlet Vert compared to the Îles de la Paix	Polluted site: The downstream sites of the St. Lawrence River (Îlet Vert, Île Beauregard and Île St. Ours) Reference sites: The upstream sites of the St. Lawrence River (Îles de la Paix, Île Dorval, Îles de Boucherville) and the Ottawa River Sampling time: June 1999–2002	[117]
Hamilton Harbour, Western Lake Ontario, Canada	White Perch	Treated domestic sewage	Intersex (22–83%) Increased Vtg levels	Polluted site: The Cootes Paradise region Reference site: Deal Lake and hatchery fish Sampling time: August 1998 and 2000, October 1999, July and September 2002	[118]
Boulder Creek and the South Platte River, Colorado, USA	White sucker	WWTP	Intersex Increased percentage of female compared to males (83% vs. 45%)	Polluted site: Boulder Creek downstream of the Boulder WWTP Reference site: A stream reach beginning 2 km upstream of the Boulder Sampling time: March May, October and November 2002	[120]
Danish Streams in Aarhus County, Jutland, Denmark	Roach	Domestic STWs	Intersex (6.7–6.5% at polluted sites compared to 4.5–5% at reference sites) The highest intersex 26.5% was observed in the stream Kristrup Landkanal.	Polluted sites: Aarhus Brook, Egaa, and Kristrup Landkanal streams receive sewage effluent discharges from STW Reference sites: Lake Almind and Lake Ravn receiving no or small amounts of STW, respectively Sampling time: June and September 1999	[119]
Boulder Creek, a Rributary of the South Platte River, Colorado, USA		WWTP that includes E_2_, EE_2_, estrone, BPA, NP	Decreased GSI Intersex (18–22%) Increased Vtg levels Decreased sperm abundance	Polluted site: Downstream of the Boulder WWTP Reference site: Upstream of the Boulder WWTP The Boulder WWTP uses filter/activated sludge treatment process with nitrification/denitrification and chlorination/dechlorination. The mean annual concentrations of NH_3_-N, NO_3_-N, biological oxygen demand, and total suspended solids in the WWTP effluent were 7, 12, 15, and 6 mg/L, respectively. At downstream of the WWTP outfall, NH_3_-N and NO_3_-N were 1 and 4 mg/L, respectively Concentrations (ng/L): E_2_ (1.6–2.1), EE_2_ (0.7–<2.0), estrone (36), BPA (2.5-35), NP (39–340) at polluted site, and E_2_ (<0.2–2.9), EE_2_ (<0.8–<2.0), estrone (<5), BPA (3.1–27), NP (<33–120) at reference site. Sampling time: Autumn 2003 and Spring 2004	[127]
Oldman, Bow and Red Deer Rivers, Alberta, Canada	Longnose dace	Municipal wastewater and agricultural runoff	Increased female-biased sex ratio at Oldman river Increased GSI at downstream of Oldman and Bow rivers Increased Vtg levels at downstream Oldman, Bow, and Red Deer rivers	Polluted sites: Downstream of Oldman, Bow and Red deer rivers (Discharges of Lethbridge, Calgary and Red Deer, respectively) Reference sites: Upstream of the above rivers Concentrations (ng/L) of selected contaminants at Red Deer and Oldman rivers: α-estradiol (0.2), E_2_ (0.6), T (1.7), Equilin (1.8), Estrone (1.8), BPA (3.8), α-Zearalanol (5.2), Ergosterol (25.4), Stigmastanol (37.5), β-sitosterol (100.5), Stigmasterol (144.7), Cholesterol (189.2), Fucosterol (212.1) Sampling time: April 2005 (Jeffries et al. 2008), Oct and No 2005 (Jeffries et al. 2010)	[123,124]
The Luvuvhu River, Limpopo Province, South Africa	Tilapia	DDT and metabolites	Decreased GSI at XW Intersex at AD, ND, and XW (55%, 60%, and 59%, respectively) Increased sperm velocity at ND Trends toward decrease in sperm motility at ND and XW	Polluted sites: Nandoni Dam (ND) and Xikundu Weir (XW) Reference site: Albasini Dam (AD) (outside of the DDT-sprayed area) Sampling surveys were carried out during high and low flow seasons Concentrations (μg/L): DDT and metabolites: 0.1–1 Sampling time: March and October 2007, February 2008	[121,122]
	African catfish		A trend toward decrease in sperm motility at ND Trends toward decrease in sperm velocity at ND and XW Testicular abnormalities at ND and XW		
The Elbe River, Czech Republic	Chub	Hg, PCBs, DDT and its metabolites, HCHs, HCB, OCS	Intersex at downstream of Usti nad Labem Decreased GSI at downstream of Pardubice and Neratovice Decreased 11-KT levels at upstream and downstream of Neratovice	Polluted sites: Downstream and upstream of Pardubice, Neratovice, and Usti nad Labem. Pardubice receives effluents of the chemical factory containing primarily active components for making medicines and pesticides. Neratovice receives effluents of the chemical plant producing caprolactam as a raw material for polyamide fiber and the making of plastics, polyvinylchloride, and several inorganic compounds. Usti nad Labem is affected by agricultural and industrial activity Reference site: The upper reaches of the Vltava River (the tributary of the Elbe River). Concentrations (mg/kg lipid): HCHs (0.021–0.486), DDT (1.14–6.48), HCB (0.109–0.521) at polluted sites and HCHs (0.018), DDT (0.92), HCB (0.033) at reference site Sampling time: May 2004	[140]
The Luvuvhu River, Limpopo Province, South Africa	Tilapia	DDT and its metabolites	Intersex at AD, ND, and XW (49%, 63%, and 58%, respectively) No intersex in the aquaria-reared fish	Polluted sites: Nandoni Dam (ND) and Xikundu Weir (XW) Reference site: Albasini Dam (AD) and reared fish	[125]
North West Province of South Africa	Catfish	DBP, NP, Se	Testicular abnormal growth in fish at HD during HF period. Trends toward decrease in sperm motility at HD during LF period Decreased sperm velocity at HD during both LF and HF periods	Polluted site: Hartbeespoort Dam (HD) characterized by eutrophication due to the high levels of phosphates and nitrates originating from STWs and agricultural effluent Reference site: Marico Bosveld Dam (MD) receiving water from the Marico River, an unmodified natural ecological state, Concentrations (μg/L): DBP: 3.5, NP: 4, Se 24, water chemistry: pH: 7.5 (MD) vs. 9–10.4 (HD), O_2_ (mg/L): 7.9 (MD) vs. 9.5–10.6 (HD), sampling time: At a low flow (LF) in Oct and at a high flow (HF) in March (MD was sampled only in Oct)	[133]
Lake Erie, Ontario, Canada	Goldfish	PCB	No difference in GSI Decreased T and 11-KT levels Decreased Vtg levels	Polluted site: Wheatley Harbour, Northwest shore of Lake Erie Reference site: Hillman Marsh	[128]
The Grand River Watershed in Southern Ontario, Canada	Rainbow darter	MWWE	Sampling time: September 2010 Decreased GSI at DS2 and DS3.5 compared to US1 Intersex at DS2 and DS3.5 compared to US1 Increased Vtg mRNA levels at DS2 compared to US1 and DS3.5 Sampling time: April and May 2011 Intersex (%): DS1 (27.3), INT1 (48.5), DS2 (84.6), DS2x (80), DS3.5 (53.3), DS Gp (6.7) vs. US1 (6.3), US2 (0), US Gp (25) No difference in GSI of normal males among US1, US2, DS1, INT1, DS2, DS2x, and DS3.5 Higher GSI of normal males at DS Gp than UP Gp No difference in GSI of intersex males No difference in E_2_ levels Decreased T levels at DS1, DS2, and DS3.5 Decreased 11-KT levels at DS1, INT1, DS2, and DS3.5 Decreased T and 11-KT levels in intersex males Decreased proportion of spermatogonia at DS2 Decreased proportion of spermatocytes and increased spermatids at INT1, DS2x, and DS3.5, no difference in proportion of sperm	Polluted site: Downstream Waterloo treatment plant outfall (DS1), between Waterloo and Kitchener MWWTPs (INT1), downstream Kitchener treatment plant outfall (DS2), downstream Kitchener outfall out of the plum. (DS2x), far downstream Kitchener treatment plant outfall (DS3.5) and downstream Guelph treatment plant outfall (DS Gp) Reference sites: Upstream urban areas (US1), upstream Waterloo outfall (US2) and upstream Guelph treatment plant outfall (US Gp) Sampling time: September 2010, April and May 2011	[129,130]
The Alibori River, Benin, West Africa	African catfish	o,p′-p,p′-DDT, o,p′-p,p′-DDE, o,p′-p,p′-DDD, endosulfan, Heptachlor	Intersex (25–50%) Decreased 11-KT levels at S3-RS, S4-RS, S3-DS Increased E_2_ levels at S2-RS, S3-RS and S4-RS, S4-DS Decreased spermatocytes, spermatids, and sperm in the testes of fish from S2-S4 compared to S1	Polluted sites: The Alibori River in Sori, Gogounou (S2), Alibori K, Kandi (S3), and Batran, Banikoara (S4). Alibori River collects the drainage from agricultural areas in the cotton-producing basin Reference site: The Pendjari River within the Pendjari National Park (S1). Agriculture is strictly forbidden Concentrations (μg/L): p,p′-DDT (0.11 RS, 0.07 DS, S4), o,p′-DDE (0.17, RS-S2, 0.19 RS-S3, 0.09 RS-S4, 0.18 DS-S2, 0.14 DS-S3, 0.17 DS-S4), p,p′-DDE, (0.13 RS-S2, 0.10 RS-S3, 0.18 RS-S4, 0.07 DS-S2, 0.08 DS-S3, 0.14 DS-S4), o,p′-DDD (0.07 RS-S2, 0.09 RS-S3, 0.09 RS-S4), p,p′-DDD (0.09 RS-S2, 0.08 RS-S4, 0.08 DS-S4), endosulfan (8.2 RS-S2, 8.8 RS-S3, 13.8 RS-S4, 2.9 DS-S2, 3.4 DS-S3, 3.7 DS-S4), Heptachlor (0.5 RS-S2, 0.5 RS-S3, 0.6 RS-S4, 1.4 DS-S2, 1.5 DS-S3, 1.3 DS-S4) Sampling time: Rainy season (RS), Sep 2010 and 2011, period of flooding with intense use of pesticides in cotton fields; dry season (DS), Feb 2011 and 2012, period of no agriculture but limited use of pesticides	[136]
	Guinean tilapia		Intersex (12.4-40%) Decreased 11-KT levels at S2-RS, S4-RS, and S4DS Increased E_2_ levels at S2, S3, and S4 both RS and DS Decreased spermatids and sperm in the testes of fish from S2–S4 compared to S1		
Grand River Watershed, Southern Ontario, Canada	Rainbow darter	MWWE	Decreased GSI at DSK and DSK2 Higher intersex at DSK, DSK2, and USK Decreased 11-KT levels at DSK, DSK2, DSW, USK, and USWDecreased T levels at DSK Increased sperm volume at DSK and DSK2 Decreased fertilization rate at DSK and USK Decreased hatching rate at DSK, USK, DSW, and USW	Polluted sites: Sites 1 km downstream of the Kitchener MWWTP (DSK) and Waterloo MWWTP (DSW), and 3 km downstream of Kitchener MWWTP (DSK2) Reference site: Sites 1 km upstream of the Kitchener MWWTP (USK) and Waterloo MWWTP (USW), and a site upstream of the city-limits (R) Sampling time: Apr and May 2012, May 2013	[134]
Lake Mead National Recreation Area, USA	Carp	DDT PCB PCP PBDEs	Decreased GSI at BB, GB, LVB and WB Decreased 11-KT levels at BB, GB, LVB, and WB Decreased E_2_ levels at BB, GB, and WB Decreased sperm motility at LVB and WB Decreased sperm viability at LVB Decreased sperm mitochondrial functions at LVB Increased sperm chromosome breakage at LVB	Polluted sites: Boulder Basin (BB), Gregg Basin (GB), Las Vegas Bay (LVB) and Willow beach (WB), Reference site: Overton Arm (OA) DDT (ng/g wet weight): 43.4 (BB), 10.5 (GB), 107.5 (LVB), 49.7 (WB), 21.3 (OA) PCBs (ng/g wet weight): 56 (BB), 14 (GB), 94 (LVB), 381 (WB), 16 (OA) PCP (ng/g wet weight): 11 (BB), 0 (GB), 82 (LVB), 0 (WB), 0 (OA) PBDEs (ng/g wet weight): 62.9 (BB), 6.4 (GB), 118.8 (LVB), 88.4 (WB), 21.1 (OA) Sampling time: May 1998, May 1999–May 2000, March–April 2006	[137]

Fish species: African catfish (Clarias gariepinus), Barbel (*Barbus plebejus),* Black bass (Micropterus spp.), Channel catfish (Ictalurus punctatus), Chub (Leuciscus cephalus), Carp (Cyprinus carpio), Chub (Leuciscus cephalus), Flounder (Pleuronectes yokohamae and *Platichthys flesus*), Goldfish (Carassius auratus), Guinean tilapia (Tilapia guineensis), Largemouth bass (Micropterus salmoides floridanus), Longnose dace (Rhinichthys cataractae), Mosquitofish (*Gambusia holbrooki*), Rainbow darter (Etheostoma caeruleum), Roach (Rutilus rutilus), Shovelnose sturgeon (Scaphirhynchus platyorynchus), Spottail shiner (Notropis hudsonius), Tilapia (Oreochromis mossambicus), White perch (Morone americana), White sucker (*Catostomus commersoni*). Hormones: 11-ketotestosterone (11-KT), 17α-ethynilestradiol (EE_2_), 17α,20β-dihydroxy-4-pregnen-3-one (17,20β-P), 17β-estradiol (E_2_), salmon gonadotropin-releasing hormone analog (D-Arg^6^,Pro^9^N-Et salmon GnRH) (sGnRH-A), luteinizing hormone (LH), testosterone (T). Chemicals: 1,1,1-trichloro-2,2-bis(p-chlorophenyl)ethane (DDT), p,p’-dichlorodiphenyldi-chloroethylene (p,p′.DDE), α-, β-, and γ-isomers of hexachlorocyclohexane (HCHs), Bisphenol A (BPA), di-n-Butyl phthalate (DBP), hexachlorobenzene (HCB), hydrophobic fragrance galaxolide (HHCB), mercury (Hg), nonylphenol (NP), octachlorostyrene (OCS), octylphenol (OP), personal care products (PCPs), polybrominated diphenyl ethers (PBDEs), polychlorinated biphenyls (PCBs), selenium (Se). Source of contaminants: Bleached kraft pulp mill effluent (BKME), municipal wastewater effluent (MWWE), sewage treatment plant (STP), sewage treatment works (STW), wastewater treatment plant effluent (WWTP).

## 6. Laboratory Evidences for Environmental Contaminants (ECs)-Related Fertility Threat in Male Fishes

Laboratory studies help us to screen the ECs adverse effects on fertility determinants to understand ECs-related fertility threat in fishes. Basically, recording male fertility at the individual level in the laboratory studies provide us with ecologically important understanding of EC-related male reproductive disorders. Moreover, there are large numbers of contaminants in the aquatic environment with agonist/antagonist interactions [141,142], which may cause difficulties to understand which kind of contaminant cause male infertility. To elucidate how each of these ECs interferes with fertility in male fishes, it is suggested to examine their effects on determinants of fertility in a single exposure protocol. This will provide valuable information on how each of these EC affects the testicular functions, which may result in diminished sperm quality.

To best of our knowledge, we found a few contaminants that have been examined over fertility tests or their adverse effects have been studied on sperm production, morphology, genome, and motility kinetics in fishes. The examined ECs could be classified into: (a) Natural or synthetic hormones (E_2_; 17*α*-ethinylestradiol, EE_2_; 17*α*-methyltestosterone, 17*α*-MT; progesterone, P), (b) alkylphenols (4-nonylphenol, 4-NP), (c) bisphenols (bisphenol A, BPA; bisphenol AF, BPAF; bisphenol S, BPS), (d) plasticizers (di-2-ethylhexyl phthalate, DEHP; mono-(2-ethylhexyl)-phthalate, MEHP), (e) pesticides (DDT and its metabolites; vinclozolin, VZ; monocrotophos, MCP; methoxychlor, DMDT), (f) pharmaceutical compounds (alderin, clofibrate, diethylstilbestrol, DES; fluoxetine, FLX; flutamide, FLU; levonorgestrel, LNG), (g) alkylating agent (methyl methane sulfonate, MMS), and (h) organotin compound (tributyltin, TBT).

In this section, we first review the laboratory studies that have investigated the adverse effects of ECs on fertility in male fishes. Then, the effects of ECs on sperm production and motility kinetics are reviewed in the laboratory studies.

### 6.1. Effects of Environmental Contaminants (ECs) on Fertility

The adverse effects of examined ECs on fertility in male fishes are summarized in Table 2. Most of these studies have shown reduction of fertilization rate or hatching rate when exposed males were mated with unexposed or exposed females, *in vivo*. Among ECs, exposure of fathead minnow (*Pimephales promelas*) to 0.1 µg/L EE_2_ and to 50 µg/L 17*α*-MT for 21 d [143,144], of medaka to 0.38 µg/L 17*α*-MT for 21 d [145], and of rainbow trout to 0.75 µg/L 4-NP for 60 d [146] resulted in complete fertility loss (Table 2). Bisphenol A [147], VZ [148], EE_2_ [149,150], and MMS [151] as high as 3120, 450, 0.5, and 50 µg/L were without effects on fertility of adult zebrafish, fathead minnow, medaka, and salmon exposed for 21, 28, 21, 21 d, respectively.

Decreases in fertility have been seen when duration of exposure of males to ECs was increased [145,146,152,153]. Additionally, increasing concentrations of a particular contaminant resulted in lower fertilization rate or hatching rate [143,145,152,153,154,155,156,157,158,159,160,161,162,163,164,165], suggesting a correlation between EC concentration and fertility. The adverse effects of a particular contaminant on male fertility show differences among species that may suggest a species- specific sensitivity to the contaminant. For instance, following exposure to 17*α*-MT for 21 d, fertilization rate is significantly decreased at 0.05 µg/L in medaka [145] and at 5 µg/L in fathead minnow [143]. If similar developmental stage in exposure test (exposure of adults or embryos to ECs) is considered, it is likely that species-specificity effects on fertilization and hatching rates exist for EE_2_ [144,149,150,155,161], BPA [147,157], and 4-NP [146,152] (Table 2).

Any changes in fertilization rate following exposure to the contaminants are similar to that of hatching rate at the same concentration [145,150,151]. This suggests a positive correlation between fertilization rate and hatching rate. Thus, fertilization rate, hatching rate, or both can be recorded to assess ECs-related male fertility in fishes.

**Table 2:** Laboratory studies on the potential adverse effects of environmental contaminants (ECs) to impair fertility in male fishes.

ECs	Fish model	Dose (µg/L)	Exposure	Fertility endpoints (%)		Description	Authors
				Fertilization Rate	Hatching Rate		
E_2_	Rainbow trout	Ctrl	Adult, 50 d	62		Sperm were prediluted in MIS^1^ and added into eggs at ratio 55,000:1 to 65,000:1. Fertilizing medium was water at 6 mL. The eyed eggs were recoded 30–35 d post fertilization.	[156] ^A^
		0.0004		59			
		0.0011		21 *			
		0.0026		7 *			
	Medaka	Ctrl	Adult, 25 d	99	76	Pairs were exposed for 21 d for females and for 25 d for males. During the exposure period, the spawned eggs were collected and the fertilized eggs (fertility of the mating pairs) were counted. During the last 3 d of exposure, the fertilized eggs from each pair were collected and the hatching rate was recorded.	[154] ^A^
		0.029		99	62		
		0.056		90	71		
		0.116		97	61		
		0.227		98	64		
		0.463		78 *	50		
EE_2_	Fighting fish	Ctrl	Adult, 28 d	78		Pairs were exposed. Nests were removed and the pairs were kept in tanks for 3 d to determine fertilization rate by counting fry numbers.	[161] ^N^
		0.01		76			
		0.1		64 *			
	Zebrafish	Ctrl	Adult, 21 d	64		Males were mated with untreated females. After 14 d, the hatching rate was calculated.	[166] ^N^
		0.025		6 *			
	Medaka	Ctrl	Adult, 21 d	98	86	Exposed spermiating male were kept in tanks with unexposed females for 7 d. All of the eggs were collected from each pair and fertilization and hatching rates were calculated.	[150] ^3^
		0.03		98	84		
		0.06		86	61		
		0.12		87	63		
		0.48		94	81		
	Fathead minnow	Ctrl	Adult, 21 d	98		Pairs were exposed. After exposure, they were transferred to aquaria with clean water. The spawned eggs were collected and the fertilized eggs (fertility of the mating pairs) were counted 2 d after spawning.	[144] ^4A^
		0.0001		89			
		0.001		92			
		0.003		84			
		0.01		30 *			
		0.1		0 *			
	Rainbow trout	Ctrl	Adult, 62 d	60 ^a^, 46 ^b^, 25 ^c^		Sperm was prediluted in MIS^1^ and added into eggs at ratio 300,000:1 (a), 50,000:1 (b), and 10,000:1 (c). Fertilization medium was ovarian fluid, 60 mM NaHCO_3_, 50 mM Tris, pH 9. The eyed eggs were recoded 28 d post fertilization	[155] ^A^
		0.016		36 ^a,^*, 23 ^b,^*,19 ^c^			
		0.131		35 ^a,^*, 22 ^b,^*,17 ^c,^*			
	Medaka	Ctrl	Adult, 21 d	92		Pairs were exposed and the spawned eggs were collected during the exposure period. The fertilized eggs (fertility of the mating pairs) were recorded.	[149] ^A^
		0.033		95			
		0.064		93			
		0.116		92			
		0.261		91			
		0.488		83			
17α-MT	Medaka	Ctrl	Adult, 21 d	99 ^a^, 98 ^b^	68 ^b^	Pairs were exposed. During the exposure period, the spawned eggs were collected and the fertilized eggs (fertility of the mating pairs) and the hatching rate were recorded following 7 d (a) or 21 d (b) of exposure.	[145] ^A^
		0.023		98 ^a^, 97 ^b^	60 ^b^		
		0.047		86 ^a^, 80 ^b,^*	39 ^b,^*		
		0.088		90 ^a^, 33 ^b,^*	18 ^b,^*		
		0.188		96 ^a^, 0 ^b,^*	0 ^b,^*		
		0.380		96 ^a^, 0 ^b,^*	0 ^b,^*		
	Fathead minnow	Ctrl	Adult, 21 d	98		After exposure, 3–4 pairs were transferred to aquaria with clean water. The spawned eggs were collected and the fertilized eggs (fertility of the mating pairs) were counted 2 d after spawning.	[143] ^5A^
		0.1		98			
		1		59			
		5		31 *			
		50		0 *			
4-NP	Medaka	0	Adult, 21 d	97	94	Pairs were exposed. For 21 consecutive d, spawned eggs were collected, counted, and assessed for fertilization rate. The fertilized eggs were maintained at the same treatments, and number of hatched embryos was recorded to assess hatching rate.	[167] ^A^
		1.27		96	98		
		2.95		97	96		
		9.81		95	99		
		27.8		95	99		
		89.4		95	77 *		
	Rainbow trout	Ctrl	Adult, 60 d		97 ^a^, 65 ^b^	Sperm was prediluted in MIS^1^ and added into eggs at ratios of 55,000:1 to 65,000:1. Fertilizing medium was water. The eyed eggs were recoded 35 d post fertilization. Fertilization was recorded following 30 d (a) and 60 d (b) of exposure.	[146] ^N^
		0.13			96 ^a^, 64 ^b^		
		0.28			94 ^a^, 68 ^b^		
		0.75			95 ^a^, 0 ^b,^*		
	Medaka	Ctrl	Adult, 21 d	98^a^, 98^b^		Pairs were exposed. During the exposure period, the spawned eggs were collected and the fertilized eggs (fertility of the mating pairs) were counted following 7 d (a) or 21 d (b) of exposure.	[152] ^A^
		24.8		98^a^, 96^b^			
		50.9		95^a^, 95^b^			
		101		98 ^a^, 89 ^b^			
		184		96 ^a^, 78 ^b,^*			
BPA	Rare minnow	Ctrl	Adult, 21 d	96 ^a,b,c^		Males were exposed to BPA for 7 d (a), 14 d (b), or 21 d (c). Fertilization was performed in vitro, and evaluated at 4 h post fertilization.	[168] ^A^
		11		92 ^a,^*, 90 ^b,^*, 89 ^c,^*			
		206		90 ^a,^*, 89 ^b,^*, 87 ^c,^*			
	Zebrafish	Ctrl	Larvae, 150 d	92	87	BPA exposure was from larvae (6 d post fertilization) to 5 months post fertilization.	[164] ^A^
		0.228		90	61 *		
		2.28		89	62 *		
		22.8		87 *	86		
	Zebrafish	0.032	Embryos, 150 d	n.s.	47	Four females and 4 males within the same group were maintained together to produce offspring for assessment of fertilization and hatching rates. Solvent contains 0.03 BPA.	[163] ^A^
		0.372		n.s.	24 *		
	Brown trout	Ctrl	Adult, 76 d	66		Sperm of exposed males was prediluted in MIS^1^ and added into eggs at ratios of 45,000:1 to 60,000:1. Fertilizing medium was water. The eyed eggs were recoded 30–35 d post fertilizat	[157] ^N^
		1.75		73			
		2.4		76			
		5.0		28 *			
	Medaka	Ctrl	Adult, 21 d	99		Pairs were exposed. During the exposure period, the spawned eggs were collected and the fertilized eggs were counted.	[147] ^A^
		837		98			
		1720		99			
		3120		99			
BPS	Zebrafish	Ctrl	Embryos, 75 d		90	Embryos were exposed to BPS. Males and females were assigned to new tanks and acclimatized for 3 d. Numbers of spawned eggs were recorded daily for the next 7 d.	[162] ^N^
		0.1			88		
		1			85		
		10			48 *		
		100			42 *		
		Ctrl	Adult, 21 d		98	Fertilized eggs of exposed female and male were collected at 16 d post exposure, and were exposed to same BPS concentrations until 6 d post fertilization, and hatching rate was determined.	[159] ^A^
		0.5			28 *		
		5			21 *		
		50			10 *		
BPAF	Zebrafish	Ctrl	Embryos, 120 d	76	99	Embryos were exposed to BPAF for 120 d. The number embryo/larvae was recorded at 7 d post fertilization.	[160] ^N^
		5		70	91		
		25		75	92		
		125		49 *	94		
MEHP	Zebrafish	Ctrl	Pre-adult (2 month-old), 81 d		82 ^a^, 82 ^b^	One hundred fertilized eggs of male and female exposed to MEHP were collected and divided into group (a) receiving the same MEHP concentrations, and group (b) receiving no further MEHP. The hatching rate was determined during 6 d post fertilization.	[165] ^A^
		0.47			82 ^a^, 54 ^b,^*		
		4.0			67 ^a^, 57 ^b,^*		
		37.5			65 ^a,^*, 47 ^b,^*		
DEHP	Zebrafish	Ctrl	Adult, 21 d		64	Males were mated with untreated females. Over a period of 14 d, the hatching rate was calculated.	[166] ^N^
		0.2			3 *		
		20			2 *		
		Ctrl	Adult, 10 d	83 ^a^, 81 ^b^		Males were injected into the intraperitoneal cavity (mg/kg). Each aquarium contains 2 females and 2 males. Following 1–5 d (a) and 6–10 d (b) of treatment, eggs were collected and fertilization rate was recorded.	[153] ^A^
		0.5		78 ^a^, 80 ^b^			
		50		88 ^a^, 78 ^b^			
		5000		75 ^a^, 48 ^b,^*			
FLU	Medaka	Ctrl	Adult, 21 d	95 ^a^, 97 ^b^, 95 ^c^		Pairs were exposed. All spawned eggs were collected from female fish and fertility of each pair were checked daily for 3 weeks: (a) 1 week, (b) 2 weeks, and (c) 3 weeks post exposure.	[169] ^A^
		100		96 ^a^, 96 ^b^, 94 ^c^			
		200		99 ^a^, 95 ^b^, 99 ^c^			
		400		94 ^a^, 100 ^b^, 95 ^c^			
		790		96 ^a^, 99 ^b^, 95 ^c^			
		1560		18 ^a,^*, 50 ^b,^*, 49 ^c,^*			
VZ	Fathead minnow	0, 60, 250. 450	Adult, 28 d	n.s.	n.s.	Pairs were exposed. The spawned eggs were collected and the fertilized eggs (fertility of the mating pairs) and hatching rate were counted during exposure.	[148] ^A^
MCP	Guppy	Ctrl	Embryos, 90 d		34	Pairs of guppies were exposed in a semi-static exposure system. Number of newly hatched offspring produced per female were counted.	[158] ^N^
		10			21 *		
		100			10 *		
		1000			9 *		
MMS	Brown trout	Ctrl	Adult, 21 d	94	93	Males were injected into the intraperitoneal cavity (mg/kg). Sperm (100 μL) were added into 40 g eggs. Fertilization medium was SAFD^2^. Fertilization rate was checked after 120 degree-days of development of eggs.	[151] ^A^
		50		95	94		
	Arctic charr	0	Adult, 21 d	84	75		
		50		83	76		

(*) Values with asterisk show statistically significant difference compared to the control. (A) Superscript of A in the Author’s column shows the actual concentrations of the contaminant. (N) Superscript of N in the Author’s column shows the nominal concentrations of the contaminant. (n.s.) No significant effects were observed. Fish species: Arctic charr (*Salvelinus alpinus*), brown trout (*Salmo trutta f. fario*), fathead minnow (*Pimephales promelas*), fighting fish (*Betta splendens*), guppy (*Poecilia reticulate*), medaka (*Oryzias latipes*), rainbow trout (*Oncorhynchus mykiss*), rare minnow (*Gobiocypris rarus*), zebrafish (*Danio rerio*). Environmental contaminants (ECs): 17α-methyltestosterone (17α-MT), 17α-ethinylestradiol (EE_2_), 17β-estradiol (E_2_), 4-nonylphenol (4-NP), bisphenol A (BPA); bisphenol AF (BPAF), bisphenol S (BPS), di-2-ethylhexyl phthalate (DEHP), flutamide (FLU), mono-(2-ethylhexyl)-phthalate (MEHP), monocrotophos (MCP), methyl methane sulfonate (MMS), vinclozolin (VZ). ^1^ Composition of the motility-inhibiting saline solution (MIS): 103 mM NaCl, 40 mM KCl, 1 mM CaCl_2_, 0.8 mM MgSO_2_, 20 mM Tris, pH 7.8. ^2^ Composition of the salmonid artificial fertilization diluent: NaCl solution, 250 mOsmol/kg, 0.05 M-glycine, 0.02 M-Tris-buffer, pH 9.0. ^3^ Actual concentrations of EE_2_: 31 and 245 ng/L (nominal 60 and 480 ng/L), respectively. ^4^ Actual concentrations of EE_2_: 0.7 and 0.8 ng/L (nominal 1 ng/L) and 8.1 and 7.8 ng/L (nominal 10ng/L) for male and female aquaria, respectively. ^5^ Actual concentrations of 17α-MT: 0.11 and 0.09 μg/L (nominal 0.1 μg/L) and 42.5 and 48.2 μg/L (nominal 50 μg/L) for male and female aquaria, respectively.

The number of sperm per an oocyte influences fertilization success, in vitro (see Section 4.1). Schultz et al. [155] examined various numbers of sperm per an oocyte to examine the effects of EE_2_ on male fertility in rainbow trout. They observed that sensitivity for detecting EE_2_ adverse effects on sperm fertilizing ability are lost at lower number of sperm per an oocyte, since the fertilization rate was low in the control group. Therefore, at first, it is essential to determine the minimum number of sperm per an oocyte to achieve an adequate fertilization rate if one uses an in vitro fertilizing assay to examine the effects of ECs on fertility.

Taken together, laboratory studies indicate that ECs are capable of reducing fertility in male fishes. However, the ECs-related fertility threat shows differences among species, and depends on concentration of the contaminant and duration of exposure. In the next sections, the biological targets through which ECs effect on male fertility are discussed.

### 6.2. Effects of Environmental Contaminants (ECs) on Sperm Production

There are a few studies that have clarified the percentage of males undergone spermiation following exposure to a contaminant (Table 3). Schoenfuss et al. [170] and Lahnsteiner et al. [157] reported significant decrease in the number of spermiating males of adult goldfish and brown trout (*Salmo trutta f. fario*) exposed to 0.05 µg/L E_2_ and 5 µg/L BPA for 70 and 76 d, respectively. McAllister and Kime [171] reported full suppression of spermiation in zebrafish males that have been exposed to 0.01 µg/L TBT from early developmental stages. These show that ECs are capable of inhibiting spermiation in male fishes.

Studies have shown reduction of sperm volume or density in male fishes exposed to E_2_ [156,170], EE_2_ [155,161], 4-NP [146], BPA [157,163,164,172,173,174], BPS [162], DEHP [175], MEHP [165], VZ [176,177,178], Flu [176,179], DDT [176], MCP [158], clofibrate acid [180], FLX [181], DES [179], and TBT [171,172] (Table 3). The adverse effects of ECs on sperm volume and density highly depend on concentrations of the contaminant. Generally, significant decreases in sperm volume or density have been observed when the concentration of contaminants or duration of exposure was increased. The effective concentration to affect sperm volume and density varies for a particular contaminant during similar duration of exposure. For examples, following 90 d of exposure, BPA significantly decreased sperm volume in goldfish at 0.2 µg/L, while sperm density was reduced at 20 µg/L [173,174]. In brown trout exposed to BPA for 38 d, sperm volume and density were decreased at 5 and 1.75 µg/L, respectively [157]. In zebrafish exposed to the metabolite of DEHP for 81 d, sperm volume and density were decreased at 37.5 and 4.9 µg/L MEHP, respectively [165]. However, a particular contaminant may affect one of the sperm production indices. For an example, in rainbow trout exposed to 0.13–0.75 µg/L 4-NP for 30 d, sperm volume was decreased at lowest dose (0.13 µg/L), while sperm density remained unchanged [146].

Laboratory studies also show that effectiveness of a contaminant on sperm production depends on duration of exposure [146,156,157,171,181]. For instances, exposure of rainbow trout to 0.001 µg/L E_2_ resulted in significant reduction of sperm volume and density following 35 and 50 d of exposure, respectively [156].

Similar to the effects of ECs on fertility, it is likely that there are species-specific effects of ECs on sperm production when similar developmental stage is considered in exposure test. For an example, when mature goldfish [174] and rainbow trout [157] were exposed to BPA, sperm volume was reduced at 0.2 and 5 µg/L, respectively. Exposure to 0.01 µg/L EE_2_ decreased sperm density in mature rainbow trout, while it was without effects on one-sided livebearer at 0.01–0.15 µg/L [155,182].

Taken together, laboratory studies indicate that ECs interfere with sperm production to affect male fertility. The degree of ECs effects depends on concentration and duration of exposure.

**Table 3:** Laboratory studies on the potential adverse effects of environmental contaminants (ECs) to impair spermiation and sperm production indices (volume and density) in male fishes.

ECs	Model	Dose (µg/L)	Exposure	Spermiation (%)	Volume (mL)	Density (×10^9^ sperm/mL)	Authors and Notes
E_2_	Grayling	Ctrl	Adult, 50 d		0.3		Authors: [156]
		0.0011			0.1 *		
	Rainbow trout	Ctrl	Adult, 50 d		4.1 ^a^, 3.8 ^b^	6.1 ^a^, 6.3 ^b^	Authors: [156]; Analysis performed 35 d (a) and 50 d (b) of exposure; Sperm volume: Sperm mass (g)
		0.0004			4.0 ^a^, 2.8 ^b^	6.0 ^a^, 6.5 ^b^	
		0.0011			2.0 ^a,^*, 1.5^b,^*	5.6 ^a^, 5.4 ^b,^*	
		0.0026			2.2 ^a^, 1.4 ^b,^*	5.7 ^a^, 3.9 ^b,^*	
	Goldfish	Ctrl	Adult, 70 d	91 ^a^, 94 ^b^			Authors: [170]; This study was performed in winter (a) and summer (b).
		0.05		67 ^a,^*, 40 ^b,^*	decrease (data not shown)	decrease (data not shown)	
EE_2_	One-sided livebearer	Ctrl	Adult, 28 d			2.4	Authors: [182]; Sperm density: ×10^6^ sperm/mL.
		0.005				2.2	
		0.064				2.2	
		0.127				2.0	
	Rainbow trout	Ctrl	Adult, 62 d			7.9	Authors: [155]
		0.016				20 *	
		0.131				30 *	
4-NP	Rainbow trout	Ctrl	Adults, 60 d		5.9 ^a^, 6.0 ^b^	7.2 ^a^, 7.0 ^b^	Authors: [146]; Analysis performed at 30 d (a) and 60 d (b) of exposure
		0.13			3.8 ^a,^*, 1.5 ^b,^*	7.4 ^a^, 6.8 ^b^	
		0.28			4.0 ^a,^*, 1.0 ^b,^*	6.9 ^a^, 6.6 ^b^	
		0.75			2.7 ^a,^*, 0.1 ^b,^*	7.3 ^a^, n.d. ^b,^*	
BPA	Rare minnow	Ctrl	Adult, 21 d			2.0 ^a^, 2.0 ^b^, 1.9 ^c^	Authors: [168]; Analysis performed at 7 d (a), 14 d (b), and 21 d (c) of exposure; Sperm density: ×10^13^ sperm/male
		11				2.0 ^a^, 2.1 ^b^, 1.9 ^c^	
		206				2.2 ^a^, 1.9 ^b^, 1.8 ^c^	
	Zebrafish	Ctrl	Embryos, 150 d		1.2	4.0	Authors: [164]; Sperm volume: ×10^7^ sperm/testis; Sperm density: ×10^9^ sperm/g testis
		0.228			1.0 *	4.0	
		2.28			1.1	4.0	
		22.8			1.2	4.0	
		Ctrl	Larvae, 150 d		1.3	4.5	
		0.228			0.8 *	3.5 *	
		2.28			1.2	4.5	
		22.8			1.3	4.5	
		Ctrl	Adult, 150 d		1.2	3.5	
		0.228			1.1	3.5	
		2.28			1.2	3.5	
		22.8			0.6 *	1.8 *	
	Zebrafish	Solvent	Embryos, 150 d			2.8	Authors: [163]; Solvent contains 0.03 BPA. Sperm density: ×10^9^ sperm/g testis
		0.37				2.2 *	
	Goldfish	Ctrl	Adult, 90 d		207.1	12.9	Authors: [173,174]; Sperm volume: μL
		0.2			52.9 *	11.1	
		2.0 20			73.3 * 58.8 *	10.8 9.2 *	
	Brown trout	Ctrl	Adult, 76 d	70 ^a^, 80 ^b^, 70^c^	0.5 ^a^, 0.5 ^b^, 0.5 ^c^	29.1 ^a^, 25.1 ^b^, 23.3^c^	Authors: [157]; Analysis performed at day 38 (a), 56 (b), and 76 (c) of exposure; Sperm volume: Sperm mass (g)
		1.75		80 ^a^, 80 ^b^, 60^c^	0.6 ^a^, 0.6 ^b^, 0.5 ^c^	26.6 ^a,^*, 26.0 ^b^, 24.1 ^c^	
		2.4		60 ^a^, 80 ^b^, 60^c^	0.6 ^a^, 0.5 ^b^, 0.7 ^c^	26.8 ^a,^*, 25.8 ^b^, 24.2 ^c^	
		5		10 ^a,^ ^b, c,^^و^	0.1 ^a,^*, 0.02 ^b,^*, 0.1 ^c^	26.2 ^a,^*, 25.2 ^b^, 25.4 ^c^	
	Guppy	Ctrl	Adult, 21 d			8.0	Authors: [172]; Sperm density: ×10^6^ sperm/mL
		274				5.1 *	
		549				2.0 *	
BPS	Zebrafish	Ctrl	Embryos, 75 d			7.8	Authors: [162]
		0.1				7.1	
		1				6.9	
		10				4.5 *	
		100				2.9 *	
FLX	Mosquitofish	Ctrl	Adult, 30 d			13	Authors: [183]; Sperm density: ×10^6^ sperm/g testis
		0.042				19 *	
		0.479				17 *	
	Goldfish	Ctrl	Adult, 14 d		23 ^a^, 0.9 ^b^		Authors: [181]; Analysis performed at 7 d (a) and 14 d (b) of exposure; Sperm volume: μL
		0.375			10 ^a,^*, 0.5 ^b^		
		45			0.7 ^a,^*,0.2 ^b,^*		
VZ	Goldfish	Ctrl	Adult, 30 d		90		Authors: [178]; Sperm volume: μL
		100			53 *		
		400			15 *		
		800			6 *		
	Guppy	Ctrl	Adult, 30 d			3.0	Authors: [177]; VZ: μg per mg dry food; Sperm density: ×10^6^ sperm/male
		0.1				2.3	
		1				1.7 *	
		10				1.0 *	
	Guppy	Ctrl	Adult, 30 d			4.9	Authors: [176]; VZ: μg per mg dry food; Sperm density: ×10^6^ sperm/male
		1				3.6	
		10				3.1	
MEHP	Zebrafish	Ctrl	Pre-adult (2 month-old), 81 d		8.3	9.8	Authors: [165]; Sperm volume: ×10^7^ sperm/d; Sperm density: ×10^10^ sperm/mL
		0.46			7.4	9.1	
		4.0			7.1	8.6 *	
		37.5			6.8 *	7.2	
DEHP	Goldfish	Ctrl	Adult, 30 d		90.4		Authors: [175]; Sperm volume: μL
		1			3.7 *		
		10			40.8 *		
		100			8.7 *		
FLU	Zebrafish	Ctrl	Adult, 30 d			1.5	Authors: [179]; Sperm density: ×10^7^ sperm/mL
		0.279				0.7 *	
	Guppy	Ctrl	Adult, 30 d			4.9	Authors: [176]; Flu: μg Flu per mg dry food Sperm density: ×10^6^ sperm/male
		1				2.9 *	
		10				1.4 *	
DES	Zebrafish	Ctrl	Adult, 30 d			1.5	Authors: [179]; Sperm density: ×10^7^ sperm/mL
		0.095				0.5 *	
TBT	Guppy	Ctrl	Adult, 21 d			8.0	Authors: 170; Sperm density: ×10^6^ sperm/mL
		0.011				3.0 *	
		0.022				2.7 *	
	Zebrafish	Ctrl	Embryos, 70 d		0.5 ^a^, 0.7 ^b^		Authors: [171]; Analysis performed at day 30 (a) and 70 (b) of exposure. At 0.01 µg/L no male was spermiated.
		0.00001			0.4 ^a^, 1.1 ^b^		
		0.001			0.6 ^a^, 2.1 ^b,^*		
		0.1			1.9 ^a,^*, 3.0 ^b,^*		
*p,p* ′-DDE	Guppy	Ctrl	Adult, 30 d			4.9	Authors: [176]; *p,p*′-DDE: μg per mg dry food; Sperm density: ×10^6^ sperm/male
		0.1				3.8 *	
		1				6.7 *	
MCP	Guppy	Ctrl	Embryos, 90 d			3.4	Authors: [158]; Sperm density: ×10^6^ sperm/mL
		10				1.9 *	
		100				2.2 *	
		1000				2.0 *	
Clofibrate	Fathead minnow	Ctrl	Adult, 21 d			4.8 ^a^, 1.2 ^b^, 1.0 ^c^	Authors: [180]; a, 53–57 females: 8–12 males; b, 6 females: 6 males, and c, 2 females: 10 males; Sperm volume is measured in ×10^6^ sperm/mg testis
		1				0.8 ^c^	
		10				0.3 ^b,^*, 0.8 ^c^	
		1000				1.7 ^a,^*, 0.5 ^b,^*, 1.1 ^c^	
MSTPs	Goldfish	Ctrl	Adult, 70 d	91^a^, 94^b^			Authors: [170]; This study was performed in winter (a) and summer (b)
		100%		100^a^, 93^b^	n.s.	n.s.	

(*) Values with asterisk show statistically significant difference from the control. (n.d.) Not determined. (n.s.) No significant difference was observed compared to control (data are not shown in the article). Fish model: Arctic charr (*Salvelinus alpinus*), brown trout (*Salmo trutta f. fario*), fathead minnow (*Pimephales promelas*), goldfish (*Carassius auratus*), grayling (*Thymallus thymallus*), guppy (*Poecilia reticulate*), medaka (*Oryzias latipes*), mosquitofish (*Gambusia holbrooki*), one-sided livebearer (*Jenynsia multidentata*), rainbow trout (*Oncorhynchus mykiss*), rare minnow (*Gobiocypris rarus*), zebrafish (*Danio rerio*). Environmental contaminants (ECs): 17α-ethinylestradiol (EE2), 17β-estradiol (E2), 4-nonylphenol (4-NP), bisphenol A (BPA), bisphenol S (BPS), di-2-ethylhexyl phthalate (DEHP), diethylstilbestrol (DES), flutamide (Flu), mono-(2-ethylhexyl)-phthalate (MEHP), monocrotophos (MCP), fluoxetine (FLX); p,p’-1,1-dichloro-2,2-bis (p-chlorophenyl) ethylene (p,p’-DDE), tributyltin (TBT), vinclozolin (VZ). Source of contaminants: Municipal sewage treatment plants (MSTPs).

### 6.3. Effects of Environmental Contaminants (ECs) on Sperm Morphology

Abnormalities in sperm morphology has been observed in zebrafish exposed to 0.0001 and 0.01 µg/L TBT for 30-70 d [171], in African catfish (*Clarias gariepinus*) exposed to 100 µg/L 4-NP for 15 d [184], in goldfish exposed to 400–800 µg/L VZ for 30 d [178], and in rare minnow (*Gobiocypris rarus*) exposed to 15 and 225 µg/L BPA [168].

The nuclear vacuoles observed in the sperm of African catfish exposed to 4-NP [184] suggest that 4-NP possess potency to damage DNA contents of sperm as there is a positive relationship between sperm nuclear vacuoles and DNA damage [185]. Recently, Zhang et al. [168] reported that number of swelling sperm was increased in rare minnow exposed to 15 and 225 µg/L BPA for two or three weeks. It is unclear whether the swelling of sperm was due to the presence of vacuoles; however, if not artifact, some vacuole-like structures were observed. Abnormalities in the sperm plasma membrane, mitochondria and flagellum were observed in sperm of zebrafish, goldfish, and rare minnow exposed to TBT, VZ, and BPA, respectively [168,171,178]. Morphological abnormalities of sperm were accompanied by decrease in the duration of sperm motility in BPA-exposed rare minnow and decreases in sperm motility or velocity in TBT-exposed zebrafish and VZ-exposed goldfish, suggesting that ECs are capable of interfering with sperm motility, as plasma membrane, mitochondria, and flagellum are involved in the osmolality-induced ATP-dependent axonemal beating of sperm (see Section 2.5).

Taken together, ECs cause abnormalities in sperm that can negatively affect sperm motility.

### 6.4. Effects of Environmental Contaminants (ECs) on Sperm Genome

In reproduction, a haploid set of the chromosomes for normal development of embryos comes from the sperm [85,186]. Sayed et al. [184] reported a decrease in the size of sperm nucleus following exposure of African catfish to 100 µg/L 4-NP. This suggests that 4-NP affected the sperm genome as a correlation exists between the nucleus size and ploidy level of sperm [86].

It has been shown that changes in the sperm chromosome number and DNA content may lead to unsuccessful fertilization or to severe genetic disease in the offspring [84,187]. Brown et al. [188] reported increases in aneuploidy sperm formation in rainbow trout exposed to 0.01 µg/L EE_2_ for 50 d. In addition, embryos propagated from exposed males to EE_2_ have shown increases in aneuploidy levels, both hypoploid (haploid) and hyperploid (triploid). Therefore, it could be speculated that observed fertility loss in rainbow trout [155] and fathead minnow [144] exposed to EE_2_ might be also related to alternations in sperm genome.

In addition to ECs-related aneuploidy sperm formation, ECs may damage DNA or cause epigenetic modifications that can shape susceptibility to disease and result in diverse phenotypes. Damage to the DNA of sperm has been reported in males of brown trout and Arctic charr following 21 d of a single intraperitoneal injection of genotoxicant MMS [151]. In this study, the author also reported aneuploidy-induced mortality and morphological abnormalities in progeny, suggesting that MMS changes in sperm genome were inherited to offspring. Corradetti et al. [166] also reported an increase in the DNA fragmentation rate in the germ cells of zebrafish exposed to 0.2 and 20 µg/L DEHP, suggesting the potential of phthalates to induce oxidative stress in the testes with consequent damage to the sperm genome.

Regarding epigenetic modifications, it has been reported that BPA affect DNA methylation in the gonads of rare minnow [189,190]. These studies have shown hypermethylation of global DNA in the testes of rare minnow exposed to 15 and 225 µg/L BPA for 7 d. The BPA-induced hypermethylation was associated with increases in DNA methyltransferase proteins that function as methylation writers and maintainers at 225 µg/L, and with decrease in ten-eleven translocation proteins that function as methylation eraser at 15 µg/L [190,191].

Taken together, these studies indicate that ECs are capable of causing damage to DNA, changing ploidy level or causing epigenetic modifications. Thus, ECs-related fertility threat might be related to alternations in sperm genome.

### 6.5. Effects of Environmental Contaminants (ECs) on Sperm Motility Kinetic

Over the past 20 years, studying sperm motility kinetics received high considerations to elucidate ECs-related fertility threat in fishes (Table 4). Studies show that sperm motility has been decreased in fish exposed to E_2_ and EE_2_ [156,170,182], 4-NP [146], BPA [157,163,164,173,174], DEHP [175], VZ [178], LNG [192], and Cu [193]. The effects of P and inclofibrate acid, alderin, FLX, and DMDT were uncertain or non-significant trends toward decrease in sperm motility were observed [180,194,195,196]. The efficiency of the contaminants to interfere with sperm motility highly depends on concentrations of the contaminants and duration of exposure. Generally, a greater decrease in sperm motility is reported when concentrations of ECs are increased (Table 4). As examples, McAllister and Kime [171] reported TBT potential to decrease and totally suppress sperm motility in zebrafish at 0.001 and 0.1 µg/L following 30–70 d of exposure from the early developmental stage, respectively. Hatef et al. [178] and Golshan et al. [175] reported that sperm motility was decreased following 30 d exposure of adult goldfish exposed to 400 and 800 µg/L VZ, and to 100 µg/L DEHP, while 100 µg/L VZ and 1 or 10 µg/L DEHP were without effects. Prolongation of the duration of exposure also results in higher effects on sperm motility. As examples, sperm motility was decreased in goldfish exposed to 4.5 µg/L BPA for 20 or 30 d, while it was not affected following 10 d of exposure [173]. Additionally, the adverse effects of a particular EC on sperm motility seems to be species-specific, if sperm motility evaluated at a similar time post initiation of motility among species with exposure test performed at similar developmental stage. For instance, exposure of adult grayling to E_2_ was reported to decrease sperm motility at 0.001 µg/L, while E_2_ was without effect on sperm motility in rainbow trout exposed to >0.001 µg/L [156]. In addition, the adverse effects of a particular contaminant are comparable in a same fish species. For an example, VZ, DEHP, and BPA reduced sperm motility in goldfish following 30 d exposure to 800, 100, and 4.5 µg/L, respectively [173,175]. In rainbow trout, significant effects of 4-NP on sperm motility were observed at 0.75 µg/L following 60 d exposure [146], while EE_2_ up to 0.1 µg/L were without effects on sperm motility following a 62 d exposure [155].

Most studies have evaluated sperm motility at one time post sperm activation. However, analyses of sperm motility at various time post activation could result in better elucidation of dose-dependent effects of ECs [173,174,175,178]. For an example, sperm motility was decreased in goldfish exposed to BPA for 90 d at 20 µg/L and 0.2 µg/L when it was evaluated at 15 and 60 s post sperm activation, respectively [174]. In another study, when sperm motility in goldfish exposed to VZ for 30 d was evaluated at 15 and 60 s post sperm activation, decreases were observed at 800 and 400 µg/L, respectively [178].

The ECs effects on sperm velocity have been shown by analyses of VCL, VSL, and VAP in the laboratory studies (Table 4). The VCL was decreased in fishes exposed to P [194], LNG [192], BPA [164,173,174], DEHP [175], VZ [178], LNG [192], clofibrate acid [180], and TBT [171]. The VSL showed significant decreases following exposure of fishes to P [194], LNG [192], BPA [164], and clofibrate acid [180]. The VAP of motile sperm were decreased in fishes exposed to E_2_ [156], P [194], and BPA [157,164].

Changes in velocity parameters identify the wave form of the flagellum of sperm during motility period. There are three models identified from the effects of ECs on sperm velocity parameters in vitro [26]. In the first model, VCL does not change, while VSL or VAP decreases. These changes have been observed in fishes exposed to E_2_ or BPA (Table 4), suggesting that sperm motility trajectory was changed from a straight direction to a circular direction. In the second model, all three parameters including VCL, VSL, and VAP are decreased. This model has been reported in fishes exposed to P, EE_2_, and BPA (Table 4), which clearly indicating changes in sperm trajectory from straight to circular. Compared to the first model, the diameter of sperm trajectories is generally smaller in the second model. There is also another model that has not been seen. The VCL decreases but VAP and VSL remain unchanged. In this case, sperm move a shorter distance over a similar period of time, while the smoothly curved direction of sperm movement remains unchanged.

Similar to the effects of ECs on sperm motility, ECs effects on sperm velocity also depend on concentrations and duration of exposure, and might be species-specific (Table 4). As an example, in adults exposure, E_2_ decreased sperm velocity in grayling at 0.001 µg/L, while it was without effects on sperm velocity in rainbow trout, goldfish and one-sided livebearer at 0.0026, 0.05, and 0.25 µg/L, respectively [156,170,197].

Similar to sperm motility, time point post sperm activation is very important to assess the effects of ECs on sperm velocity. For an example, VCL at 15 s post sperm activation was not differed in goldfish exposed to 0.6–11 µg/L BPA for 10, 20, or 30 d, while VCL showed a significant decrease when it was evaluated at 60 s post sperm activation [174].

Taken together, these studies indicate ECs effects on sperm motility and velocity, suggesting that ECs-related diminished fertility in males might be due to decreases in sperm motility kinetics.

**Table 4:** Laboratory studies on the potential adverse effects of environmental contaminants (ECs) to impair sperm motility kinetics in male fishes. Motility shows percentage of motile sperm evaluated at time post activation (TPA) following activation of sperm motility in an activation medium (AM). Sperm velocity characters are the curvilinear velocity (VCL), the straight line velocity (VSL), and the angular path velocity (VAP). Some studies have used an immobilizing medium (IM) to prevent spontaneous initiation of sperm motility at stripping or to predilute sperm before analysis of the motility.

ECs	Model	Dose (µg/L)	Exposure	TPA (s)	Motility (%)	Velocity (µm/s)			Authors and Notes
						VCL	VSL	VAP	
E2	One-sided livebearer	Ctrl	Adult, 28 d	240 (every 10 s)	79	113	110		Authors: [197]; AM: HAMF-10 medium
		0.05			78	115	111		
		0.10			70	115	111		
		0.25			75	118	116		
	Rainbow trout	Ctrl	Adult, 50 d	10	89			78	Authors: [156]; IM (mM): 103 NaCl, 40 KCl, 1 CaCl_2_, 0.8 MgSO_2_, 20 Tris, pH 7.8; AM (mM): 60 NaHCO_3_, 20 Glycin, pH 9.0; Analysis done at 35 d post exposure.
		0.0004			87			82	
		0.0011			85			84	
		0.0026			80			90	
	Grayling	Ctrl	Adult, 50 d	10	74			105	Authors: [156]; IM and AM: See above row
		0.001			46 *			78 *	
	Goldfish	Ctrl	Adult, 70 d	n.d.	2500 ^a^, 3000 ^b^		100 ^a^, 62 ^b^		Authors: [170]; IM: Ringer solution; AM: DW; Motility was absolute number of motile sperm per fish. Exposures were performed in winter (a) and summer (b).
		0.05			<10 ^a,^*, <10 ^b,^*		112 ^a^, 75 ^b^		
P	Fathead minnow	Ctrl	Adult, 7 d	80 (every 15 s)	53	94	75	90	Authors: [194]; IM: 0.8 mM NaCl solution. AM: DW
		0.025			63.5	78	52	73	
		0.339			39	60 *	38 *	53 *	
EE_2_	Zebrafish	Ctrl	Embryos, 240 d	44 (every 2 s)	58	89	56		Authors: [198]; IM: n.d.; AM: n.d.; Solvent: DMSO
		Solvent			49	80	47		
		0.0002			53	87	49		
		0.00024			38	79	49		
		0.001			47	88	52		
	one-sided livebearer	Ctrl	Adult, 28 d	240 (every 10 s)	82	103	101		Authors: [182]; AM: HAMF-10 medium
		0.005			75	109	105		
		0.064			76	107	103		
		0.127			65 *	106	109		
	Fighting fish	Ctrl	Adult, 28 d	60 (every 6 s)					Authors: [161]; IM (g/L): 5.5 NaCl, 2 KCl, 3.8 Glycine, 2.4 Tris, pH 7.5; AM: Water containing EE_2_
		0.01			n.s.	n.s.	n.s.	n.s.	
		0.1			n.s.	n.s.	decrease	n.s.	
	Medaka	Ctrl	Adult, 21 d	30		70	60		Authors: [150]; AM (g/L): 1 NaCl, 0.03 KCl, 0.04 CaCl_2_, 0.1 MgCl_2_, 0.2 NaHCO_3_, pH 7.3
		0.06				88	85		
		0.12				85	83		
		0.24				90	75		
		0.48				83	81		
	Rainbow trout	Ctrl	Adult, 62 d	12 (every 15 s)					Authors: [155]; IM (mM): 103 NaCl, 40 KCl, 1 CaCl_2_, 0.8 MgSO_2_, 20 Tris, pH 7.8; AM (mM): 60 NaHCO_3_, 50 Tris, pH 9
		0.016			n.s.	n.s.	n.s.		
		0.131			n.s.	n.s.	n.s.		
LNG	Fathead minnow	0	Adult, 14 d	80 (every 15 s)	85	68	81	52	Authors: [192]
		0.012			74 *	60	62 *	48	
		0.127			45 *	47*	58 *	46	
BPA	Rare minnow	Ctrl	Adult, 21 d	n.d.	97 ^a, b, c^				Authors: [168]; IM: Hank’s balance; AM: 0.55% NaCl
		11			97 ^a^, 97 ^b^, 96 ^c^				
		206			97 ^a^, 96 ^b^, 95 ^c^				
	Zebrafish	Ctrl	Embryos, 150 d	n.d. (15 frames each 0.05 s)	65	128	74	87	Authors: [164]; IM: Hank’s balance; AM: n.d.
		0.228			53*	120 *	58 *	73*	
		2.28			61	113 *	50 *	68*	
		22.8			61	116	62	76	
		Ctrl	Larvae, 150 d		68	128	66	86	
		0.228			55 *	128	66	84	
		2.28			61	117 *	62	76	
		22.8			62	120	58 *	72 *	
		Ctrl	Adult, 150 d		65	130	74	87	
		0.228			60	125 *	68	84	
		2.28			59	125 *	62 *	76 *	
		22.8			45 *	125 *	60 *	76 *	
	Zebrafish	Solvent	Embryos, 150 d	n.d. (15 frames each 0.05 s)	75	100			Authors: [163]; IM: Hank’s balance; AM: n.d. Solvent contains 0.03 BPA
		0.37			36 *	68			
	Goldfish	Ctrl	Adult, 30 d	15	97 ^a^, 100 ^b^, 99 ^c^	157 ^a^, 146 ^b^, 166 ^c^			Authors: [173];AM (mM): 50 NaCl, 20 Tris, pH 8.5, osmolality 110 mOsmol/kg; Analyses performed at day 10 (a), 20 (b), and 30 (c) of exposure
		0.6			94 ^a^, 94 ^b^, 95 ^c^	148 ^a^, 156 ^b^, 159 ^c^			
		4.5			96 ^a^,80 ^b,^*,88 ^c,^*	158 ^a^, 136 ^b^, 158 ^c^			
		11.0			98 ^a^, 85 ^b,^*,91 ^c,^*	144 ^a^,138 ^b^, 154 ^c^			
		Ctrl		60	90 ^a^, 79 ^b^,	55 ^a^, 30 ^b^, 38 ^c^			
		0.6			78 ^a^, 74 ^b^, 70 ^c,^*	34 ^a,^*, 25 ^b,^*, 30 ^c,^*			
		4.5			85^a^, 61^b,*^, 68^c,*^	29 ^a,^*, 34 ^b,^*, 38 ^c,^*			
		11.0			85 ^a^, 65 ^b,^*, 61 ^c,^*	30 ^a,^*, 29 ^b,^*, 37 ^c,^*			
	Goldfish	Ctrl	Adult, 90 d	15	99	168			Authors: [174]; AM (mM): 50 NaCl, 5 KCl, 20 Tris, pH 8.5, osmolality 110 mOsmol/kg
		0.2			94	155			
		2			95	150 *			
		20			88*	140 *			
		Ctrl		60	64	60			
		0.2			51 *	59			
		2			46 *	48			
		20			41 *	47 *			
	Brown trout	Ctrl	Adult, 76 d	10	84 ^a^, 88 ^b^, 86 ^c^			97 ^a^, 107 ^b^, 106 ^c^	Authors: [157]; IM (mM): 103 NaCl, 40 KCl, 1 CaCl_2_, 0.8 MgSO_2_, 20 Tris, pH 7.8; AM (mM): 60 NaHCO_3_, 20 Glycin, pH 9.0; Analyses were performed at day 38 (a), 56 (b), and 76 (c) of exposure
		1.75			33 ^a,^*, 73 ^b^, 70 ^c^			62 ^a,^*, 75 ^b,^*, 111 ^c^	
		2.4			40 ^a,^*, 36 ^b,^*, 81 ^c^			75 ^a,^*, 75 ^b,^*, 92 ^c^	
		5			4 ^a,^*, 1 ^b,^*, 4 ^c^			32 ^a,^*, 49 ^b,^*, 41 ^c,^*	
4-NP	Rainbow trout	Ctrl	Adult, 60 d	10	68 ^a^, 75 ^b^, 69 ^c^			82 ^a^, 92 ^b^, 89 ^c^	Authors: [146]; IM (mM): 103 NaCl, 40 KCl, 1 CaCl_2_, 0.8 MgSO_2_, 20 Tris, pH 7.8; AM (mM): 60 NaHCO_3_, 20 Glycin, pH 9.0; Analyses were performed at day 0 (a), 30 (b), and 60 (c) of exposure
		0.13			69 ^a^, 70 ^b^, 65 ^c^			77 ^a^, 96 ^b^, 84 ^c^	
		0.28			72^a^, 75^b^, 81^c^			81^a^, 101^b^, 78^c^	
		0.75			66^a^, 70^b^, n.d.^c,*^			82^a^, 91^b^, n.d.^c,*^	
FLX	Mosquitofish	Ctrl	Adult, 35 d		85	112	62	80	Authors: [196]; IM (mM): 207 NaCl, 5.4 KCl, 1.3 CaCl2, 0.49 MgCl2, 0.41 MgSO4, 10 Tris, pH 7.5; AM: 150 mM KCl with 2 mg/mL BSA
		0.031			83	115	65	84	
		0.375			84	108	61	77	
VZ	Goldfish	Ctrl	Adult, 30 d	15	99	165			Authors: [178]; AM (mM): 50 NaCl, 20 Tris, pH 8.5 Osmolality 110 mOsmol/kg
		100			97	159			
		400			80	137			
		800			19^*^	130^*^			
		Ctrl		60	64	64			
		100			52	58			
		400			44^*^	65			
		800			14^*^	60			
DEHP	Goldfish	Ctrl	Adult, 30 d	15	98	161			Authors: [175]; AM (mM): 50 NaCl, 5 mM KCl. 20 Tris, pH 8.5
		1			86	142			
		10			88	128*			
		100			74*	107*			
		Ctrl		60	67	67			
		1			46*	60			
		10			47*	63			
		100			38*	68			
Clofibrate	Fathead minnow	Ctrl	Adult, 21 d	5	62	30	22		Authors: [180]; IM (mM): 94 NaCl, 27 KCl, 50 Glycine, 15 Tris pH. 7.6; AM: Water; Sperm velocity in nm/s.
		1			56	26	16		
		10			51	24	18		
		1000			50	21 *	11 *		
TBT	Zebrafish	Ctrl	Fry, 70 d	5		78 ^a^, 60 ^b^			Authors: [171]; IM (g/L): 5.8 NaCl, 0.2 KCl, 0.2 CaCl_2_, 0.04 MgCl_2_, 2.1 NaHCO_3_, 0.04 NaH_2_PO_4_, 3.8 glycine, pH 8.6; AM: DW; Analyses were performed at day 30 (a) and 70 (b) of exposure
		0.00001				62 ^a^, 62^b^			
		0.001				44 ^a,^*, 19 ^b,^*			
		0.01				n.d. ^a,^*, 20 ^b,^*			
		0.1				36 ^a,^*, 0 ^b,^*			
MSTPs	Goldfish	Ctrl	Adult, 70	n.d.	2500 ^a^, 3000 ^b^		100 ^a^, 62 ^b^		Authors: [170]; IM: Ringer solution; AM: DW; Sperm motility shows absolute number of motile sperm per fish. Exposures were performed in winter (a) and summer (b)
		100%			2000 ^a^, 2700 ^b^		100 ^a^, 95 ^b^		
Cu	Killifish	Ctrl	Fry, 345 d		83				Authors: [193]; Ctrl contains 1.8 Cu
		7.1			26 *				
		10.9			17 *				
Alderin	Catfish	0			75	109	89	103	Authors: [195]; IM (g): 5.49 NaCl, 2.01 KCl, 3.75 glycine, 1.82 Tris; AM: Tank water
		0.14			71	100	80	91	
DMDT		0.23			68	86	71	81	

(*) Values with asterisk show statistically significant difference from the control. (DW) distilled water. (n.d.) Not determined. (n.s.) No significant difference was observed compared to control (data are not shown in the article). Environmental contaminants (ECs): 17α-ethinylestradiol (EE_2_), 17β-estradiol (E_2_), 4-nonylphenol (4-NP); bisphenol A (BPA), copper (Cu), di-2-ethylhexyl phthalate (DEHP), fluoxetine (FLX), methoxychlor (DMDT), levonorgestrel (LNG); progesterone (P), tributyltin (TBT), vinclozolin (VZ). Municipal sewage treatment plants, STPs; fish models: African catfish (*Clarias gariepinus*), brown trout (*Salmo trutta f. fario*), fathead minnow (*Pimephales promelas*), fighting fish (*Betta splendens*), goldfish (*Carassius auratus*), grayling (*Thymallus Thymallus*), killifish (*Poecilia vivipara*), medaka (*Oryzias latipes*), mosquitofish (*Gambusia holbrooki*), one-sided livebearer (*Jenynsia multidentata*), rainbow trout (*Oncorhynchus mykiss*), rare minnow (*Gobiocypris rarus*), zebrafish (*Danio rerio*). Source of contaminants: Municipal sewage treatment plants (MSTPs). Chemicals: Bovine serum albumin (BSA).

**Table 5:** Environmental contaminants (ECs)-related male fertility endpoints at environmentally relevant concentrations. The lowest and highest tested concentrations are shown as LTC and HTC, respectively.

ECs	Environmental Concentration (ng/L)	Fish Species	LTC (ng/L)	HTC (ng/L)	Fertility	Sperm			Authors
						Production	Motility	Velocity	
E_2_	0.1–5.0^1^, 0.2–2.9^2^, 0.6^3^, 0.5–5.2^4^, 0.4–3.3^5^, 0.3–55.0^6^, 2.7–48.0^7^, LOD-3.7^11^, 7.48^20^, LOD-7.4^26^, LOD-84.3^27^, LOD-33.4^28^, <1–175^29^	Zebrafish	-	25	+	n.d.	n.d.	n.d.	[166] ^N^
		One-sided livebearer	50	250	n.d.	n.d.	-	-	[197] ^A^
		Rainbow trout	0.4	2.6	+	+	-	-	[156] ^N^
		Medaka	29	463	-	n.d.	n.d.	n.d.	[154] ^A^
		Grayling	-	1.0	n.d.	+	+	+	[156] ^N^
		Goldfish	-	50	n.d.	+	+	-	[170] ^A^
P	7.4–11.8^1^, 5.4–6.1^8^	Fathead minnow	25	339	n.d.	n.d.	-	-	[194] ^A^
EE_2_	0.7–<2.0^2^, 0.1–8.9^4^, 0.04^5^, 0.2–7.5^9^, LOD-42.0^10^, LOD-0.8^11^; LOD-35.6^28^, <0.8–34.0^29^	Fighting fish	10	100	-	n.d.	-	-	[161] ^N^
		Zebrafish	0.2	1	n.d.	n.d.	-	-	[198] ^A^
		Medaka	30	480	-	n.d.	n.d.	-	[150] ^A^
		Fathead minnow	0.1	100	+	n.d.	n.d.	n.d.	[144] ^1^
		Rainbow trout	16	131	+	+	-	-	[155] ^A^
		Medaka	33	488	-	n.d.	n.d.	n.d.	[149] ^A^
		One-sided livebearer	10	150	n.d.	-	-	-	[182] ^A^
17α-MT	1.3–1.8^8^, <0.9–14.5^12^, 1.33^13^	Medaka	23	380	-	n.d.	n.d.	n.d.	[145] ^A^
		Fathead minnow	100	50,000	-	n.d.	n.d.	n.d.	[143] ^2^
4-NP	LOD-37,000^11^, LOD-10,186^14^, 15–386^15^, 77–1142^16^, 112.4–2065.7^26^, <0.5–211.0^29^	Medaka	1,270	89,400	-	n.d.	n.d.	n.d.	[167] ^A^
		Rainbow trout	130	750	+	+	+	+	[146] ^N^
		Medaka	248	184,000	-	n.d.	n.d.	n.d.	[152] ^A^
BPA	LOD-12,205^14^, LOD-1125^16^, LOD-8000^17^, 0.5–702^18^, 6.6–74.9^19^, 12.3–755.6^26^, <1–145^29^	Rare minnow	11,000	206,000	+	-	-	n.d.	[168] ^A^
		Zebrafish	228	22,800	+	+	+	+	[163,164] ^A^
		Brown trout	1,750	5,000	+	+	+	+	[157] ^N^
		Medaka	837,000	3,120,000	-	n.d.	n.d.	n.d.	[147] ^A^
		Goldfish	200	20,000	n.d.	+	+	+	[173] ^A^, [174] ^N^
		Guppy	274,000	549,000	n.d.	-	n.d.	n.d.	[172] ^N^
BPS	0.3–19.0^19^	Zebrafish	100	100,000	-	-	n.d.	n.d.	[162] ^N^
		Zebrafish	500	50,000	-	+	n.d.	n.d.	[159] ^A^
BPAF	LOD-15,000^43^	Zebrafish	5000	125,000	-	n.d.	n.d.	n.d.	[160] ^N^
DEHP	330–182,000^18^, 230–730^20^, 364–2.68^21^, 61.6–4352.0^22^, 150–12,100^41^	Zebrafish	200	20,000	+	n.d.	n.d.	n.d.	[166] ^N^
		Zebrafish	500	5,000,000	-	n.d.	n.d.	n.d.	[153] ^A^
		Goldfish	1000	100,000	n.d.	+	+	+	[175] ^N^
VZ	0.5–20^23^, 5^24^, <10^25^	Fathead minnow	60,000	450,000	-	n.d.	n.d.	n.d.	[148] ^A^
		Goldfish	100,000	800,000	n.d.	-	-	-	[178] ^N^
		Guppy	100	10,000	n.d.	-	n.d.	n.d.	[177] ^A^
		Guppy	1000	10,000	n.d.	-	n.d.	n.d.	[176] ^A^
DES	LOD-3.3^27^, LOD-8.5^28^	Zebrafish	-	95	n.d.	-	n.d.	n.d.	[179] ^A^
FLX	0.4–2.6^30^, 2.0–19.5^31^; 4.7–9.4^32^; LOD-128^33^	Mosquitofish	42	479	n.d.	+	-	-	[183] ^A^, [197] ^A^
		Goldfish	375	45,000	n.d.	±	n.d.	n.d.	[181] ^A^
FLU	0.55–1.1^34^, 12–30^44^	Zebrafish	-	279	n.d.	±	n.d.	n.d.	[179] ^A^
		Medaka	100,000	1,560,000	-				[169] ^A^
		Guppy	1000	10,000	n.d.	±	n.d.	n.d.	[176] ^A^
Clofibrate	LOD-17.2^32^; 0.2–0.7^35^, 6–7000^44^	Fathead minnow	1000	1,000,000	n.d.	±	-	-	[180] ^A^
MCP	8.3^36^, 165^37^, LOD-4000^38^	Guppy	10,000	1,000,000	±	±	n.d.	n.d.	[158] ^N^
MMS	0.1^39^	Brown trout	-	50,000	-	-	n.d.	n.d.	[151] ^N^
		Arctic charr	-	50,000	-	-	n.d.	n.d.	[151] ^N^
TBT	0.42^40^	Guppy	11	22	n.d.	±	n.d.	n.d.	[172] ^N^
		Zebrafish	0.01	100	n.d.	±	n.d.	±	[171] ^N^
DDE	360^42^	Guppy	100	1000	n.d.	+	n.d.	n.d.	[176] ^A^
Aldrin	140^42^	African catfish		140	n.d.	n.d.	-	-	[196] ^N^
DMDT	230^42^	African catfish		230	n.d.	n.d.	-	-	[196] ^N^

(+) A significant decrease was observed at environmental relevant concentration. (–) No significant decrease was observed at the maximum tested concentration of the contaminant. (+/–) The lowest examined dose was higher than environmental relevant concentration, however significant decrease was observed. (A) Superscript of A in the Author’s column shows the actual concentrations of the contaminant. (N) Superscript of N in the Author’s column shows the nominal concentrations of the contaminant. (n.d.) Not determined. (LOD) Lower of Detection. Environmental contaminants (ECs): 17α-ethinylestradiol (EE_2_), 17α-methyltestosterone (17α-MT), 17β-estradiol (E_2_), 4-nonylphenol (4-NP); bisphenol A (BPA), Bisphenol AF (BPAF), Bisphenol S (BPS), *p,p*′-1,1-dichloro-2,2-bis (*p*-chlorophenyl) ethylene (*p,p*′-DDE), Di-2-ethylhexyl phthalate (DEHP), Diethylstilbestrol (DES), Flutamide (FLU), Fluoxetine (FLX), Methoxychlor (DMDT), Monocrotophos (MCP), Progesterone (P), Tributyltin (TBT), Vinclozolin (VZ). References for environmental concentrations of the contaminants: 1. Velicu and Suri [199]; 2. Vajda et al. [127]; 3. Jeffries et al. [123]; 4. Kuch and Ballschmiter [200]; 5. Baronti et al. [201]; 6. Nasu et al. [202]; 7. Desbrow et al. [203]; 8. Liu et al. [204]; 9. Belfroid et al. [205]; 10. Ternes et al. [206]; 11. Snyder et al. [207]; 12.Yu et al. [208]; 13. Blankvoort et al. [209]; 14. Höhne and Püttmann [210]; 15. Cailleaud et al. [211]; 16. Mohapatra et al. [212]; 17. Kang et al. [213]; 18. Fromme et al. [214], 19. Yang et al. [215]; 20. Bókony et al. [216]; 21. Wen et al. [217]; 22. Zhang et al. [218]; 23. Reedman et al. [219]; 24. Kreuger et al. [220]; 25. Zheng et al. [221]; 26. Wang et al. [222]; 27. Chen et al. [223]; 28. Lei et al. [224]; 29. Pojana et al. [225]; 30. Wu et al. [226]; 31. Paíga et al. [227]; 32. Lindim et al. [228]; 33. Sanots et al. [229]; 34. Yan et al. [230]; 35. Nentwig et al. [231]; 36. Anjum and Malik [232]; 37. Kang and Zhang [233]; 38. Kumari et al. [234]; 39. Canty et al. [235], 40. Guo et al. [236]; 41. Li et al. [237]; 42. Nibamureke et al. [238]; 43. Song et al. [239]; 44. Corcoran et al. [240]. Fish species: African Catfish (*Clarias gariepinus*), Arctic charr (*Salvelinus alpinus*), Brown trout (*Salmo trutta f. fario*), Fathead minnow (*Pimephales promelas*), Fighthing fish (*Betta splendens*), Goldfish (*Carassius auratus*), Grayling (*Thymallus Thymallus*), Guppy (*Poecilia reticulate*), Medaka (*Oryzias latipes*), Mosquitofish (Gambusia holbrooki), One-sided livebearer (*Jenynsia multidentata*), Rainbow trout (*Oncorhynchus mykiss*), Rare minnow (*Gobiocypris rarus*), Zebrafish (*Danio rerio*). ^1^ Actual concentrations of EE_2_: 0.7 and 0.8 ng/L (nominal 1 ng/L) and 8.1 and 7.8 ng/L (nominal 10 ng/L) for male and female aquaria, respectively. ^2^ Actual concentrations of 17α-MT: 0.11 and 0.09 μg/L (nominal 0.1 μg/L) and 42.5 and 48.2 μg/L (nominal 50 μg/L) for male and female aquaria, respectively.

## 7. Conclusions

**Figure 5:**
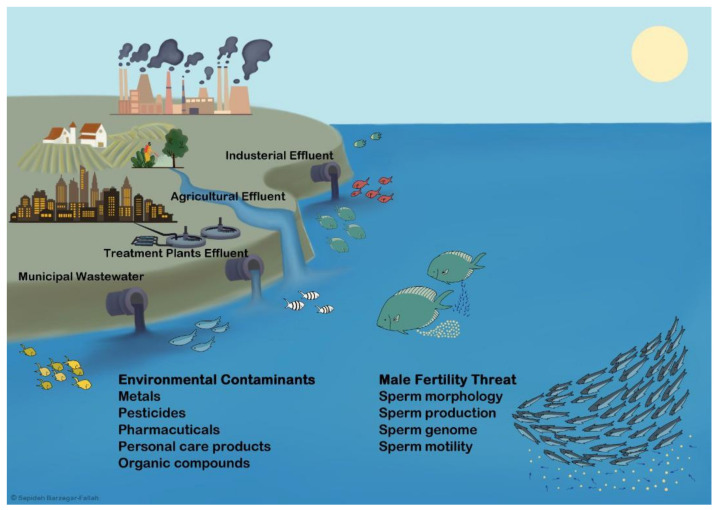
The adverse effects of environmental contaminants (ECs) on fertility in male fishes. The aquatic environments are the final repository of the municipal wastewater, and industrial, agri- cultural, and treatment plants effluents that contain various natural and man-made contaminants including metals, pesticides, pharmaceuticals, and organic compounds, as well as compounds used in personal care products. Evidences from wildlife and results of laboratory studies reveal adverse effects of ECs on sperm production, morphology, genome, and motility to cause fertility threat at the level of the individual. Contribution of ECs to declining fish populations is largely unknown and needs to be elucidated.

Both wildlife and laboratory studies show the adverse effects of ECs on fertility in male fishes. However, the sites of action of ECs to reduce male fertility are highly diverse. They may reduce sperm production, cause damage to sperm morphology, alter the sperm genome, or decrease sperm motility and velocity (Figure 5).

It has been shown that in vitro exposure of semen (or sperm) to ECs causes damage to sperm and decreases sperm motility and velocity to decline sperm fertilizing ability; however, the effective concentrations are far exceeding the WHO recommended limits [26]. The present review on laboratory studies highlights that adverse effects of ECs on determinants of fertility (including sperm production, morphology, genome, and motility kinetics) can appear at environmentally relevant concentrations when male fishes are exposed to ECs in vivo (Table 5). These support the hypothesis that diminished fertility in fishes from wildlife might be related to the contaminants in the aquatic environment.

The present review shows that: (a) Unlike the source of contaminants from industrial, municipal, treatment plant, or agricultural activities, abnormalities in sperm morphology [137,139], damage to the sperm genome [137], decrease in sperm production (volume or density) [115,117,127,132,136], decrease in sperm motility or velocity [115,117,121,122,132,133,137], and diminished male fertility [115,132,134] have been observed in fishes from wildlife that were associated with delay in sexual maturation, decrease in testicular size, histological defects in the testis, and increase in female bias sex ratio and intersex (Table 1). (b) The laboratory studies reveal that ECs reduce male fertility in concentration-dependent and exposure time-dependent manners. There is at least one study that shows E_2_, EE_2_, 17*α*-MT, 4-NP, BPA, BPS, and DEHP and its metabolite (MEHP) cause diminished fertility at environmentally relevant concentration (Table 2 and Table 5). (c) The ECs-related diminished fertility in males at environmentally relevant concentrations is associated with the adverse effects of ECs on at least one of the determinants of fertility. There is at least one study that shows the adverse effects of almost all examined ECs on sperm production, morphology, genome, or motility kinetics at environmentally relevant concentrations (Table 3, Table 4 and Table 5). It is worth to note that both wildlife and laboratory studies [13,17,18,19,20,21,22,23,24,25] suggest that the adverse effects of ECs on the determinants of fertility that cause diminished fertility were associated with alternations in hormonal functions of HPT and increased circulating Vtg levels (Table 1). However, our aim in the present study was not to review the effects of ECs on hormonal functions of HPT.

## 8. Future Research Directions

The present review highlights the following aspects that should be taken into account in further studies to better elucidate the ECs-related fertility threat in male fishes.

**Figure 6:**
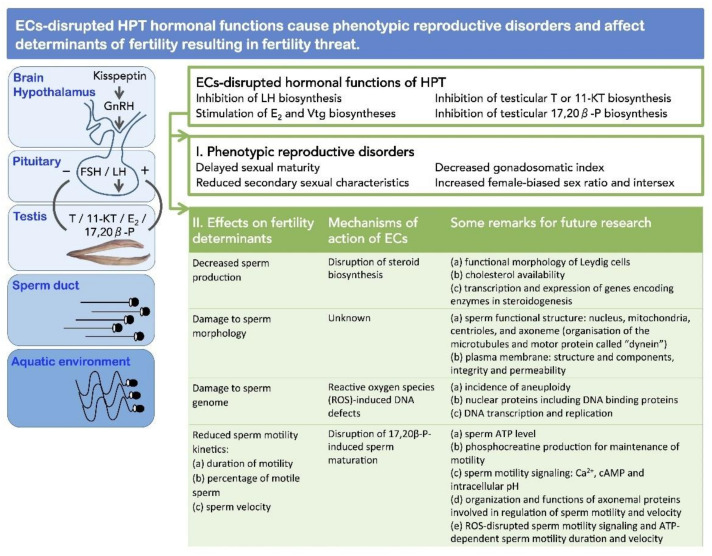
Effects and mechanisms of action of environmental contaminants (ECs) on the reproductive system and fertility in male fishes. In the left, hypothalamus–pituitary–testis (HPT) regulation of sperm production and maturation is shown. The follicle-stimulating hormone (FSH) regulates 11-ketotestosterone (11-KT)-stimulated spermatogenesis. At spawning, luteinizing hormone (LH) regulates 17*α*,20*β*-dihydroxy-4-pregnen-3-one (17,20*β*-P)-stimulated sperm maturation. Sperm are released into the sperm ducts to acquire potential for the motility initiation. In the right, lessons from wildlife and laboratory studies on the reproductive system in male fishes are shown. ECs cause phenotypic changes in the reproductive system and affect determinants of fertility. It has been reported that adverse effects of ECs on sperm production, genome, and motility kinetics are associated with generation of reaction oxygen species (ROS) and inhibition of testicular testosterone (T), 11-KT, and 17,20*β*-P. GnRH, gonadotropin-releasing hormone; E_2_, 17*β*-estradiol; Vtg, vitellogenin.

(a) One can easily distinguish that the number of contaminants that have been used in fertility tests are comparable to the number of contaminants in our environment. Future studies should examine the effects of other chemicals with major public concerns. Among them, there is an immediate need to examine the effects of pharmaceuticals (such as antidepressants, antibiotics, antidiabetics, and contraceptives) that are highly consumed in global medicine use, agriculture, and animal husbandry [24,241]. Fertilizers and biocides (such as antimicrobial compounds and pesticides), personal care products (such as antifungal agents and cosmetics), and industrial contaminants (such as heavy metals, plasticizers and polycyclic aromatic compounds) are other types of contaminants that need immediate consideration [242,243,244].

(b) Examination of the adverse effects of ECs at environmentally relevant concentrations that cover the lowest and highest detected concentrations is highly needed. Most studies have failed to include the lowest environmental concentration among treatments (Table 5).

(c) The ontogeny-dependent effects of the ECs on male fertility are largely unknown. Basically, developmental stages are the main parameters to be carefully defined based on the objective. For instance, if the study is aiming at investigating the adverse effect of ECs on sperm production, it is a prerequisite to expose the fish from the early developmental stage of the testis. However, the present review shows that a number of studies that exposed fishes to ECs at early life stages is considerably lower than those that examined the adverse effects of ECs in adult-exposed individuals (Table 2, Table 3 and Table 4). When embryos are exposed, the study reports the adverse effects of ECs throughout the life, which is close to the physiological condition in the environment. It is worth to note that it has been hypothesized that the effects of ECs is high at the early developmental stage due to the fact that alternations in normal developmental and physiological phenomena are irreversible [245]. One of the key advantages and significant reasons for studying the adverse effects of ECs at different developmental stages is to elucidate the condition in which a fish species displays migration within its life cycle. In this regard, we could find only one study that compared the adverse effects of ECs at different developmental stages [164]. The authors reported that the adverse effects of BPA on sperm production and number of spermiated males were highly differed if embryos, larvae, or adults were exposed (Table 3 and Table 4).

(d) Assessment various types of determinants of fertility to better elucidate site of action of an EC that cause diminished fertility. There are a few studies that have evaluated the ECs effects on sperm production and motility (Table 5).

(e) The present study shows that ECs may cause phenotypic changes in the reproductive system and affect determinants of fertility (Figure 6). It has been well known that ECs disrupts hormonal regulation of spermatogenesis to decrease sperm production [17,20,23,25,27]. However, our knowledge on mechanisms of action of ECs on sperm morphology, genome, and motility are largely unknown.

To better elucidate ECs effects on sperm morphology, future studies should investigate functional structure of sperm including nucleus, mitochondria, centrioles, and axoneme, with particular consideration to the organization of the microtubules and motor protein called “dynein” in the latter case [168,171,178,184,246,247]. Studying structure and components, integrity, and permeability of the sperm plasma membrane can help us to better understand association between ECs-induced abnormalities of sperm and its motility kinetics.

Regarding the ECs effects on sperm genome, it is needed to examine effects of ECs on incidence of aneuploidy [188], to study nuclear proteins including DNA binding proteins and DNA transcription and replication using omics tools [246], and to study sperm epigenetic functions (see Section 8).

Regarding sperm motility, there are a few studies that show that the ECs effects on sperm motility kinetics might be related to a decrease in the ATP source required for flagellar beating or to damage to sperm morphology [135,161,168,171,178,184,247]. However, the effects of ECs to interfere with intracellular messengers (such as pH, Ca^2+^, and cAMP) that regulate sperm motility signaling are fully unknown and should be considered in future studies [246]. Schiffer et al. [248] showed that ECs alters intracellular Ca^2+^ through activation of a Ca^2+^ channel to interfere sperm motility in human. The laboratory studies suggest that ECs may affect sperm swimming pattern as sperm velocity parameters are decreased (Table 4). Future researches may investigate organization and functions of axonemal proteins involved in regulation of sperm motility and velocity as well as generation of reactive oxygen species (ROS) that may disrupt sperm motility signaling and may affect sperm velocity.

(f) Sperm requires energy for motility and flagellar beating force [66,68,106]. Our knowledge on the adverse effects of ECs on ATP content of sperm is very limited. Montgomery et al. [161] reported a decrease in sperm ATP content when a fighting fish (*Betta splendens*) was exposed to 0.1 µg/L EE_2_, which was associated with decrease in VSL. This suggests that the adverse effects of EE2 on sperm velocity could be related to amount of ATP available to generate flagellar beating force. Further studies are needed to investigate sperm energetics along with sperm motility kinetics to better understand the mechanisms through which ECs cause fertility threat.

However, assessment of the adenylate energy charge (AEC = ATP + 0.5 ADP/ATP + ADP + AMP) is suggested to better understand the adverse effects of ECs on sperm energetics and metabolism of energy [26,66,249]. Moreover, ECs may affect phosphocreatine production, which is necessary for maintenance of motility [246].

(g) In an aquatic environment, there are mixtures of ECs that may exhibit pharmacodynamic interactions. These interactions can be antagonistic or synergistic, which may cause an increase in the toxicity of a particular chemical through the cocktail effect [141,142]. For instance, Schoenfuss et al. [170] reported no changes in sperm production, motility, and velocity when goldfish adults were treated with municipal sewage treatment plants. However, they observed significant decrease in sperm production and motility when the goldfish were exposed to E2. Therefore, another very interesting issue that requires an immediate consideration is to examine the interaction effects of various contaminants in combination exposure tests. The results will also provide significant information to better understand the mechanistic effects of ECs on fertility.

(h) Our current knowledge on the ECs-related male fertility threat is mainly from freshwater fishes inhabiting lakes or rivers (Table 1). Future wildlife and laboratory studies should sample from the primitive fishes as well as marine fishes due to their significance in ecological impacts in the aquatic environment (such as fishes of coral reefs), biological conservation program (such as sturgeons and eels), or in commercial fisheries (such as ornamental fishes).

(i) Recently, epigenetic inheritance of the adverse effects of ECs received high scientific and public considerations. As ECs have been shown to affect the sperm genome, further studies need to characterize transgenerational inheritance of the adverse effects of ECs to the offspring. There are studies that show the adverse effects of bisphenols and 4-NP that were inherited to the next generations [163,167]; however, it is largely unknown for the other ECs. Chen et al. [163] reported that male offspring of adult zebrafish that have been exposed to BPA from early developmental stages showed a decrease in sperm production, while no effects on sperm motility or fertility were seen. These were reported for the offspring grown in BPA-free water. However, growing of offspring of zebrafish in the same concentrations of BPA resulted in decreases in sperm production, motility, and fertility, similar to their parents that were exposed to BPA from early developmental stages.

(j) The threat of ECs to fish populations is still an open question. There are historical reports that show populations of fishes have declined [250,251,252]; however, the contributions of ECs were not known. Hamilton et al. [252] suggested linking exposure and fish health at the population level, examining combinations of ECs, and studying the implications of population-genetic methods to address the adverse effects of ECs on fish populations. On their comments, the establishment of a long-term study that uses a model species from wildlife, investigates chronic effects of ECs, considers genetic diversity, and elucidates fertility within individuals of the population is needed. Moreover, development of an ecological habitat (such as artificial river) where a small fish population is routinely exposed to ECs in situ would be a great help.

## 9. Suggestions to Normalize Future Studies

We also would like to offer the following suggestions to normalize the studies for better understanding of the ECs-related fertility threat in fishes.

(a) The large variations among results of studies that have examined a particular contaminant suggest the need to optimize a protocol for harmonization of studies, which should encompass both biological and technical issues. These may include a protocol for optimizing the design of exposure tests, methods for evaluating male fertility, and laboratory tools to assess determinants of fertility. For example, (a-i) CASA has become very popular for the analyses of sperm motility kinetics. Technical conditions for recording of sperm motility and setting of CASA affect the results [253], thus need to be standardized for laboratory models. (a-ii) Most laboratory studies have used distilled water and/or activation medium to assess sperm motility kinetics (Table 4). To approach real effects of ECs, future studies should use the water of the aquarium where the fish were exposed to EC. (a-iii) Analysis of sperm motility kinetics needs to be performed at earliest time post sperm activation. It would be also useful if studies considered similar time periods to better understand the risk assessment of ECs. In this regard, analysis of sperm motility kinetics within 10 to 30 s post activation is recommended, as (a) one can operate the microscope and CASA easily [106] and (b) sperm fertilizing ability highly decreases after 30 s post activation [104]. (a-iv) As another example, indices of sperm production (sperm volume and density) could be expressed as total or normalized to body weight [28,106].

(b) The present study suggests species-specific adverse effects of an EC on male fertility and its determinants. To better understand sensitivity of a species to an EC, the effects of ECs could be examined on small laboratory fishes including zebrafish, guppy and medaka, which serve as aquatic model organisms. Application of species with different biology of reproduction will provide us with valuable information to assess ecological risk assessment of ECs. Among species, zebrafish and Medaka, in contrast to guppy, are external fertilizers, and the testes are of the tubular type in zebrafish and guppy compared to those of medaka that are of the lobular type.

(c) Although all studies have considered this note, it is worth reminding that one should take into account renewal of exposure medium based on the half-life of the EC.

## Data Availability

All data analyzed for the present study are included in this published article.
